# Nodal modulator (NOMO) is a force-bearing transmembrane protein required for muscle differentiation

**DOI:** 10.1083/jcb.202505010

**Published:** 2025-07-15

**Authors:** Brigitte S. Naughton, Swapnil C. Devarkar, Vanessa Todorow, Sunanda Mallik, Stacey Oxendine, Sanjana Junnarkar, Yuan Ren, Julien Berro, Janine Kirstein, Yong Xiong, Christian Schlieker

**Affiliations:** 1 Department of Molecular Biophysics and Biochemistry, Yale University, New Haven, CT, USA; 2 National Institutes of Health Post-Bac Research Education Program for Biological & Biomedical Sciences, Yale University, New Haven, CT, USA; 3 Nanobiology Institute, Yale University, West Haven, CT, USA; 4 Department of Cell Biology, Yale School of Medicine, New Haven, CT, USA; 5 Leibniz Institute on Aging – Fritz-Lipmann-Institute, Jena, Germany; 6 Friedrich-Schiller-Universität, Institute for Biochemistry and Biophysics, Jena, Germany

## Abstract

The ER relies on membrane-shaping proteins to maintain a continuous network of sheets and tubules that host distinct biological processes. How this intricate structure of the ER membrane system is maintained under conditions of mechanical strain is incompletely understood. NOMO is an ER-resident transmembrane protein that contributes to ER morphology and is highly expressed in striated muscle. In this study, we identify a critical interface between distal Ig domains that enables NOMO to maintain ER morphology and bear mechanical forces. By incorporating two independent tension sensors in the luminal domain of NOMO, we demonstrate that NOMO assemblies experience forces in the single piconewton range, with a significant contribution from the identified interface. These newly defined features are important—if not indispensable—for myogenesis, as interface mutations affecting mechanosensitivity fail to restore the essential role of NOMO during myogenesis in a C2C12 differentiation model. Moreover, NOMO depletion impairs nematode motility, underscoring a broader functional importance in muscle physiology.

## Teaser

Naughton et al. establish that the ER experiences forces in the piconewton range across the ER lumen and identify NOMO as a force-bearing transmembrane protein that is required for myogenesis.

## Introduction

Nodal modulator (NOMO) (also known as pM5) encodes a highly conserved type I transmembrane protein implicated in vertebrate development and ER structural integrity (Haffner et al., 2004; Zhang et al., 2015; Zhao et al., 2017; Amaya et al., 2021). While only one copy exists in other organisms, three nearly identical paralogs (NOMO1, NOMO2, and NOMO3; hereafter referred to as NOMO) are encoded in the human genome, each featuring a large ER luminal domain (LD), a single transmembrane domain, and a short cytosolic tail, with a total mass of ∼139 kDa. NOMO was first studied in zebrafish for its role in attenuating nodal signaling during embryonic cellular differentiation (Haffner et al., 2004; Shen, 2007). It associates with a variety of ER-resident proteins or protein complexes, including nicalin (Haffner et al., 2004), which interacts with TMEM147 (Dettmer et al., 2010), a resident of the back of Sec61 complex implicated in multi-pass membrane protein biogenesis (Sundaram et al., 2022; Page et al., 2023, *Preprint*). Ectopic expression of NOMO1 and nicalin in zebrafish embryos induces cyclopia (Haffner et al., 2004), and NOMO is significantly downregulated in patients with facial asymmetry associated with skeletal malocclusion (Nicot et al., 2014). NOMO is highly expressed in heart and skeletal muscle (Uhlen et al., 2015) and reduced in patients with ventricular septal defect (Zhang et al., 2006), a common congenital heart anomaly, signaling a contribution to cardiovascular health and disease.

These findings collectively suggest that NOMO plays a multifaceted role in both developmental and disease processes. Nevertheless, the cellular and molecular mechanisms underlying these observations are poorly understood. Further research into NOMO functional pathways is essential to elucidate disease etiology and potential therapeutic targets. In our previous work, we reported that NOMO depletion causes large fenestrations in the ER, whereas overexpression constricts ER sheets to an intermembrane spacing that scales with the molecular dimensions of NOMO’s LD (Amaya et al., 2021). However, the functional relevance in relation to molecular features of NOMO, particularly in muscle tissue and in an organismal context, remained unexplored.

In this study, we integrate AlphaFold predictions and structural comparisons to identify an interface between distal NOMO Ig folds that we validate through in vitro reconstitution and determine to have modest affinity. These low-affinity interfaces are critical for the establishment of metastable NOMO assemblies that exhibit unusually slow diffusion dynamics within the ER. By applying two independent tension sensors (TSs), we demonstrate that NOMO assemblies experience intraluminal forces in the piconewton (pN) range. The force-bearing features identified in this study are crucial for NOMO’s mechanosensitive properties during myogenesis, as shown by the necessity of interface-proficient NOMO for myocyte differentiation in a C2C12 model. Lastly, silencing of the NOMO homolog nicotinic acid receptor protein-4 (*nra-4*) in *Caenorhabditis elegans* significantly reduces the motility of the nematode. Collectively, our observations suggest an unexpected role for NOMO as an ER-resident mechanosensitive protein, with implications for understanding NOMO-linked developmental disorders affecting skeletal muscle and heart function.

## Results

### NOMO comprises conserved Ig-like domains similar to those of force-bearing proteins

**Figure 1.**
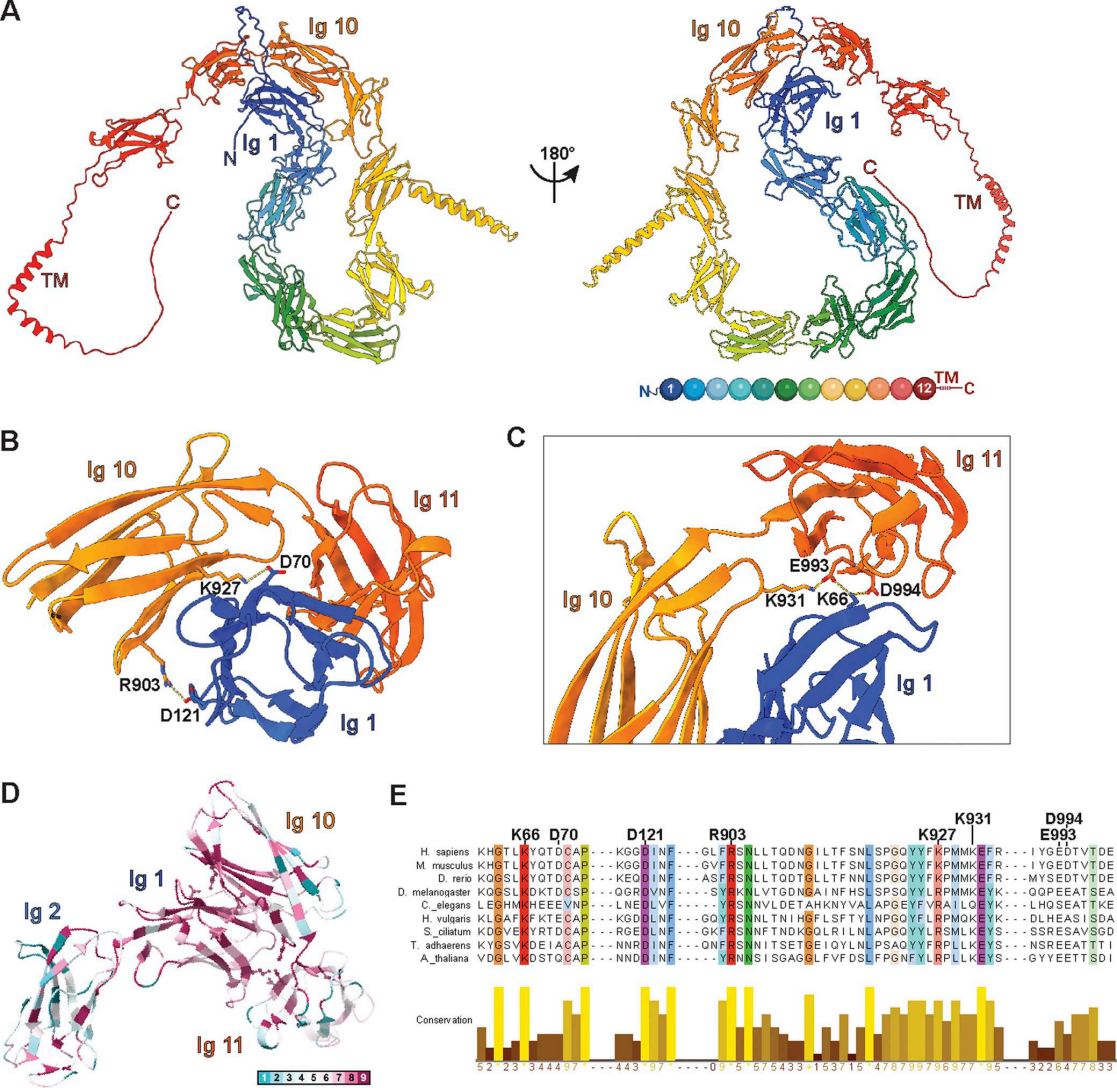
**Predicted structural features of NOMO. (A)** An AlphaFold3 model prediction of FL human NOMO1 resolves twelve Ig-like folds, colored by Ig domains depicted as balls in the bottom right cartoon. NOMO1 is represented in two views related by a 180° rotation, as indicated. **(B)** Magnification of the interface formed by Ig 1/10/11 with D70-K927 and D121-R903 salt bridges labeled. **(C)** An alternative view of the Ig 1/10/11 interface with K66-D994-K931-E993 salt bridges labeled. **(D)** Ig 1–2 and Ig 10–11 interface conservation. Inset, ConSurf color-coding scheme from (1) variable to (9) conserved. **(E)** Sequence conservation of regions around and including the salt bridges shown in B and C, colored by >80% conservation. UniProt ID: Q15155.

**Figure S1.**
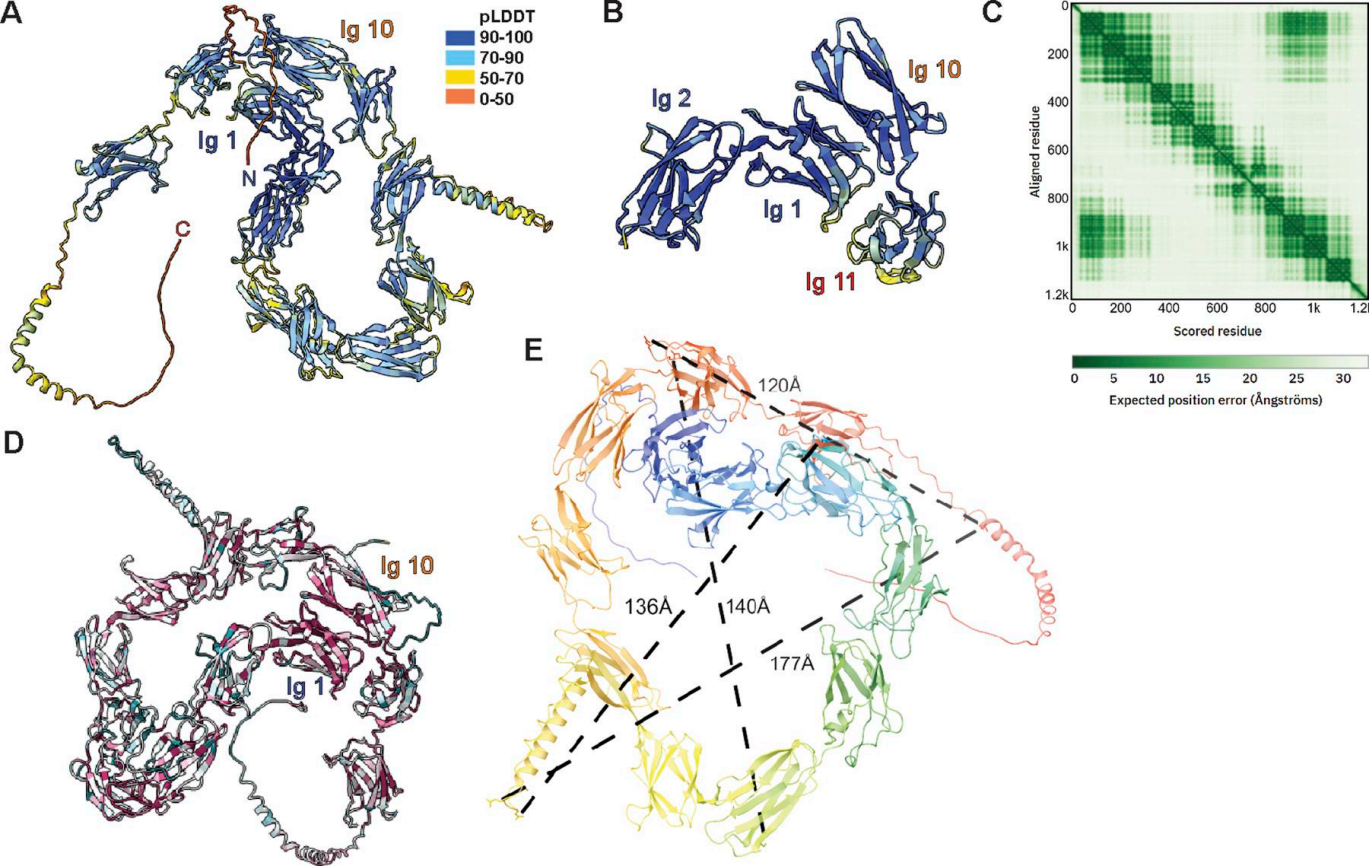
**NOMO structure predictions are confident and conserved. (A)** AlphaFold3 models a highly confident structure of NOMO, colored by the per-residue confidence metric predicted local distance difference test (pLDDT) on a scale from 0 to 100, depicted in the legend inset. **(B)** Ig 1–2 and Ig 10–11 colored by pLDDT. **(C)** NOMO predicted aligned error (PAE) measuring the confidence of the relative position of two residues within the AlphaFold2 structure plot. **(D)** NOMO protein sequence conservation as predicted by ConSurf. Color scale is the same as in Fig. 1 E. **(E)** Representative distances in Å between distal regions in NOMO.

To gain insights into NOMO’s architecture, we previously purified and subjected full-length (FL) NOMO1-FLAG to negative-stain EM, which revealed a flexible, extended rod-like structure ∼27 nm in length (Amaya et al., 2021). Two-dimensional classifications resolved a “beads on a string” morphology with eight discernible globular segments, possibly representing Ig-like domains. However, the flexibility of NOMO posed experimental obstacles to resolving the FL structure. High-confidence AlphaFold3 predictions (pTM = 0.54) (Abramson et al., 2024) of NOMO (Fig. 1, A–D and Fig. S1, A–E) have since corroborated the presence of 12 Ig-like domains that reside in the ER lumen, followed by a single transmembrane domain and a short cytosolic tail (Fig. 1 A). The model posits NOMO as adopting a looping topology facilitated by an interface formed by distal Ig domains, consistent with the PAE plot (Fig. S1 C). Within this interface, we identified several salt bridge interactions involving Ig 1 D70–Ig 10 K927, Ig 1 D121–Ig 10 R903, Ig 11 D994,E993–Ig 1 K66, and Ig 10 K931 (Fig. 1, B and C). Consistent with an important role for this interface, residues contributing to this interface are evolutionarily conserved (Fig. 1, D and E and Fig. S1 D) (Ashkenazy et al., 2016; Waterhouse et al., 2009).

### Salt bridge interactions are critical for NOMO function and impart slow diffusional mobility on the NOMO assembly

**Figure 2.**
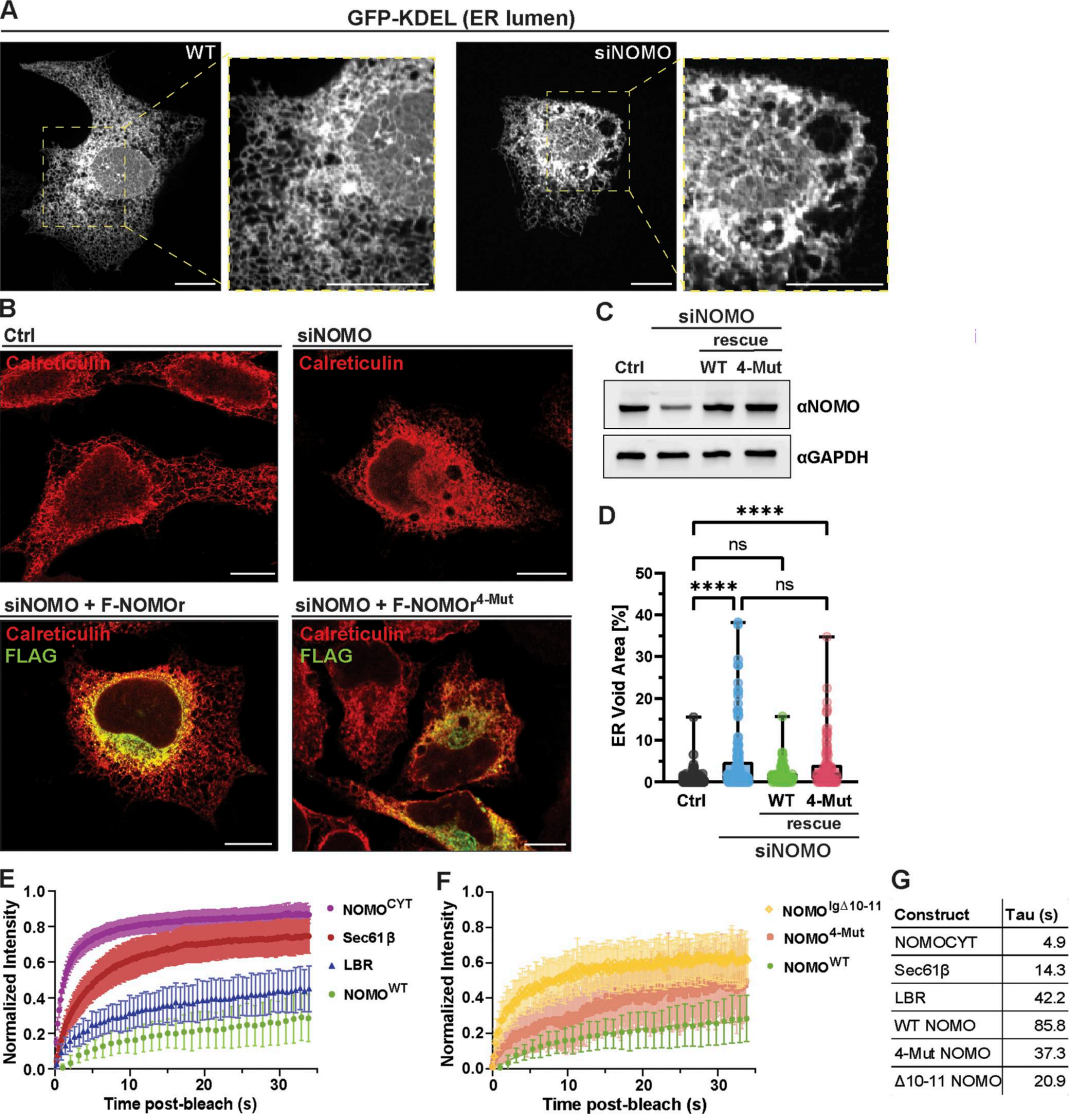
**NOMO relies on the predicted Ig 1/10/11 interface for functionality. (A)** Spinning disc confocal images of U2OS cells treated with either non-targeting (WT) or NOMO-targeting (siNOMO) RNAi. **(B)** Representative immunofluorescence images of U2OS cells treated with non-targeting siRNA (Ctrl) or NOMO-targeting siRNA (siNOMO), with indicated rescue constructs transfected in lower panels. **(C)** Immunoblot of cell lysates from experimental setups as in B. **(D)** Plot of mean ± SD of ER voids area normalized to total ER area determined by calreticulin staining under indicated conditions; *n* = 131 cells (Ctrl), *n* = 137 cells (siNOMO), *n* = 116 cells (siNOMO + F-NOMOr), and *n* = 115 cells (siNOMO + F-NOMOr^4-Mut^). **(E and F)** FRAP data showing means ± SD of fluorescence intensities of bleached regions normalized to the pre-bleach intensity for each condition. *n* ≥ 30 cells across a *N* ≥ 3 biological replicates. Note that NOMO^WT^ data are the same in E and F. **(G)** Summary of mean tau (τ) values for each condition corresponding to FRAP experiments shown in E and F. Statistical analyses were performed using an ordinary one-way ANOVA test; ****P < 0.0001 and ns, not significant. Scale bars, 10 μm. Source numerical data and unprocessed blots are available in source data. Source data are available for this figure: SourceData F2.

**Figure S2.**
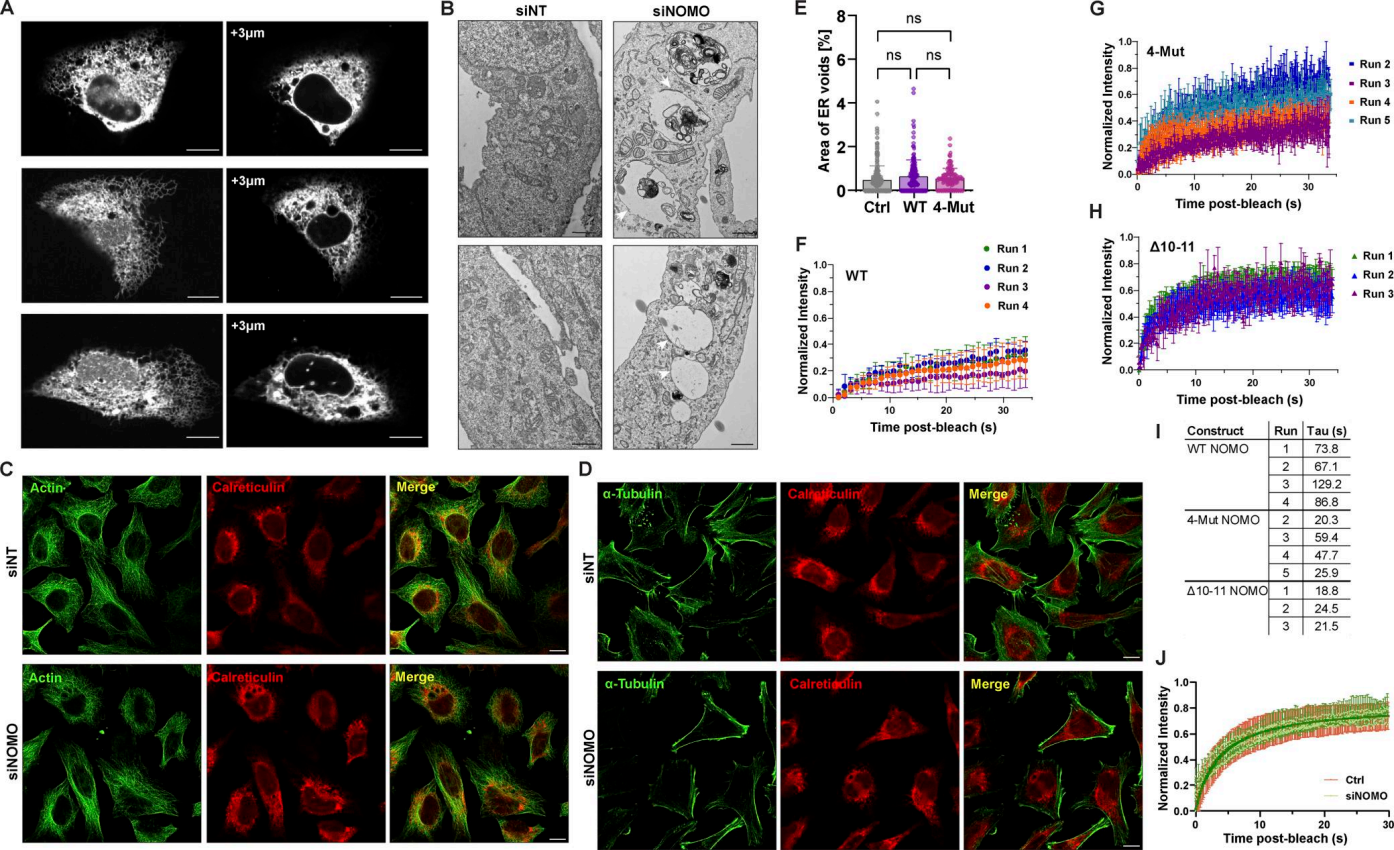
**NOMO depletion disrupts ER architecture and dynamics. (A)** Spinning disc confocal microscopy images of U2OS cells under NOMO knockdown (siNOMO), showing z-stack projections at +3 µm from the basal plane shown at the left. Scale bars: 10 µm. **(B)** Transmission electron microscopy (TEM) images of control (siNT) and NOMO knockdown (siNOMO) U2OS cells. Arrows (black) denote ER; white arrowheads denote membrane delineated structures “voids” arising upon NOMO depletion. Scale bars: 1 µm. **(C and D)** Immunofluorescence images of HeLa cells under either non-targeting (siNT) or NOMO knockdown (siNOMO) conditions with calreticulin and the indicated cytoskeletal stain. **(E)** Quantification of void area in WT U2OS cells across conditions (Ctrl, WT NOMO, and 4-Mut NOMO). Data represent mean ± SD. Statistical significance was determined by an ordinary one-way ANOVA test; *ns*, not significant. **(F–H)** Superplots of FRAP data for WT NOMO, 4-Mut NOMO, and Δ10–11 NOMO constructs, with multiple biological replicates (Runs) displayed with a minimum of 10 cells per run. Each plot represents normalized fluorescence intensity over time after bleaching. **(I)** Table of FRAP recovery tau values (τ, in seconds) for indicated NOMO constructs across multiple experimental runs from E. **(J)** FRAP analysis of ER-localized Sec61β -GFP in control (Ctrl) and NOMO knockdown (siNOMO) cells. Source numerical data are available in source data.

The ER of WT U2OS cells forms an extensive and intricate network of tubules and sheets that spread throughout the cell, with reticular tubules predominantly in the peripheral regions and dense sheets predominantly near the nucleus (Fig. 2 A, left). Upon NOMO depletion, membrane-delineated voids appear in the ER network, ranging from <0.5 to 5 µm (Fig. 2 A and Fig. S2 A). These voids often contain membranous species (Fig. S2 B), do not stain for typical ER-resident proteins, and appear to have minimal impact on the surrounding cytoskeletal organization in the cytoplasm (Fig. S2, C and D). Overexpression of an siRNA-resistant WT NOMO1 construct with an N-terminal Flag tag (F-NOMOr) rescues the NOMO depletion phenotype (Fig. 2, B and D), as scored by the area of the total ER occupied by voids, assessed by calreticulin staining.

To assess whether the Ig 1 and Ig 10–11 interface (Ig 1/10/11) is important for NOMO function, we engineered an siRNA-resistant NOMO1 construct with four charge-reversal mutations predicted to disrupt the interface (K66E, D70K, D121K, and K931E, designated F-NOMOr^4-Mut^). F-NOMOr^4-Mut^ expression failed to rescue the void phenotype in NOMO knockdown conditions (Fig. 2, B and D). Immunoblotting of cell lysates from each condition confirmed efficient depletion of endogenous NOMO and similar expression of both F-NOMOr and F-NOMOr^4-Mut^ (Fig. 2 C). When we expressed F-NOMO or F-NOMO^4-Mut^ in WT U2OS cells expressing endogenous NOMO, we found that neither construct significantly induced a void phenotype (Fig. S2 E).

We next examined NOMO and ER-resident protein dynamics in the ER by employing FRAP in HeLa cells. ER-resident Sec61β with a GFP tag (Sec61β -GFP) served as a highly diffusive control (Shibata et al., 2008), and nuclear-resident GFP-tagged lamin B receptor (GFP-LBR) served as a slowly diffusive control (Fig. 2 E). F-NOMOr was tagged with an internal eGFP-tagRFP moiety (TS) between Ig 12 and the TM domain (NOMO-TS_in_), explained in detail below. We additionally included a TS-tagged soluble C-terminal tail of NOMO (CYT) with rapid recovery kinetics to ensure the TS moiety did not interfere with mobility. We photobleached eGFP with a 1-µm diameter circular ROI in either the ER (for Sec61β and NOMO) or nucleus (for LBR) and monitored recovery over time. In contrast to the rapid diffusion of Sec61β (τ = 14 s), NOMO exhibited markedly slow and restricted lateral mobility (τ = 86 s) (Fig. 2, E–G). Notably, NOMO’s recovery profile was even slower than that of LBR (τ = 42 s), which displays limited mobility due to its attachment to the nuclear lamina (Ellenberg et al., 1997; Ungricht et al., 2015).

To test whether the Ig 1/10/11 influences NOMO’s diffusional dynamics, we transfected F-NOMO^4-Mut^ and a variant with Ig 10–11 removed via an in-frame deletion (F-NOMO^Δ10–11^), each containing an internal TS at the same position as the WT construct. Removing either the entire ∼20-kD region corresponding to Ig 10–11 or introducing the four-point mutations at the Ig 1/10/11 interface increased NOMO mobility (F-NOMO^Δ10–11^, τ = 21 s and F-NOMO^4-Mut^, τ = 37 s) (Fig. 2, F and G; and Fig. S2, F–I). Collectively, these FRAP data indicate that NOMO diffuses slowly within the ER membrane, relying in part on the presence of the Ig 1/10/11 interface. However, the absence of NOMO does not appear to significantly affect global protein movement in the ER membrane, as evidenced by normal Sec61β -GFP mobility under NOMO knockdown (Fig. S2 J).

### Ig 1–2 and Ig 10–11 dimerize in vitro, and their expression induces a dominant-negative void phenotype

**Figure 3.**
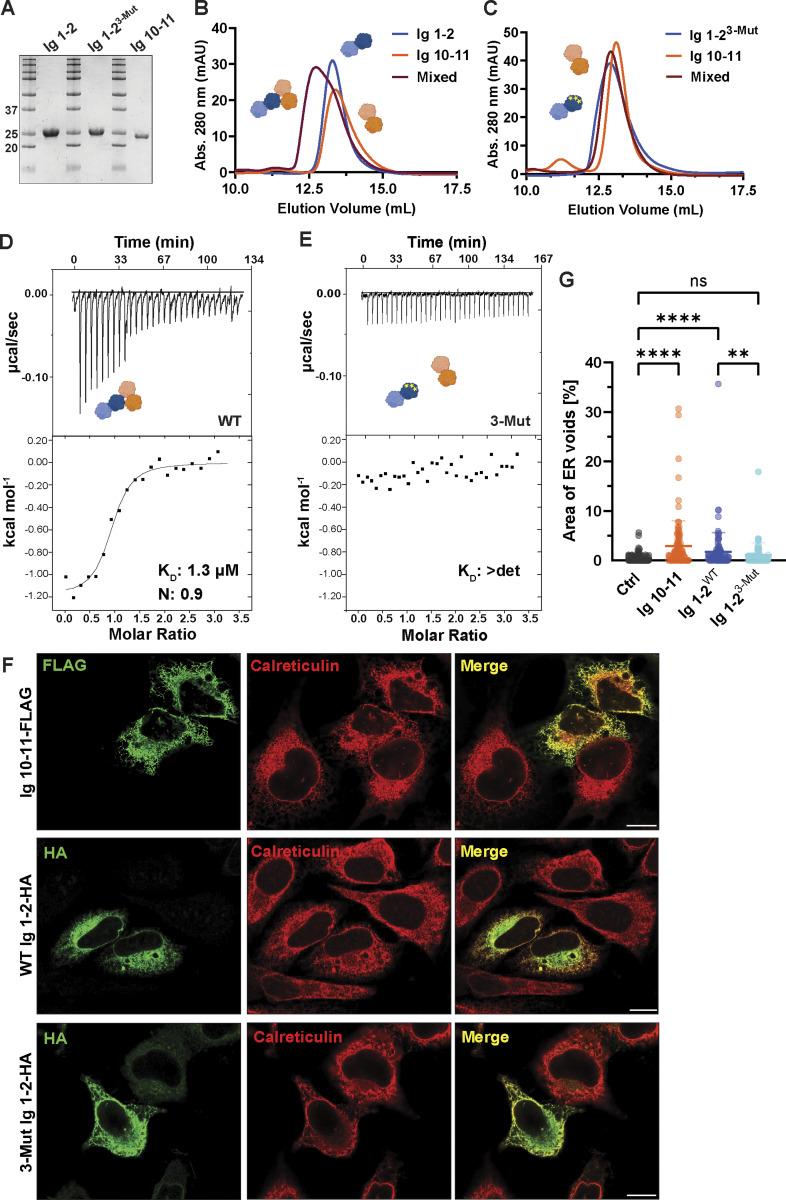
**Ig 1**–**2 and Ig 10**–**11 dimerize and induce ER voids upon overexpression. (A)** SDS-PAGE/Coomassie staining of constructs obtained from preparative SEC. **(B and C)** SEC profiles of recombinantly purified Ig 1–2 and Ig 10–11 on a HiLoad Superdex 75 pg. **(D and E)** ITC-binding studies of constructs used in A–C with dissociation constants (K_D_) and binding stoichiometry (*n*) indicated. *n* = 3 biological replicates. **(F)** Representative U2OS cells immunostained for the ER marker calreticulin and for either Ig 1–2-HA or Ig 10–11-FLAG expression. **(G)** Plot of mean void area relative to ER area per cell scored for the indicated construct expression. *N* = 113 untransfected cells (Ctrl), *N* = 103 Ig 10–11-FLAG–transfected cells, *N* = 105 WT Ig 1–2-HA–transfected cells, and *N* = 107 3-Mut Ig 1–2-HA–transfected cells from. Error bars denote ± SD. Statistical analyses were performed using an ordinary one-way ANOVA test; ****P < 0.0001, **P < 0.01, and ns, not significant. Scale bars, 10 μm. Source numerical data and unprocessed blots are available in source data. Source data are available for this figure: SourceData F3.

**Figure S3.**
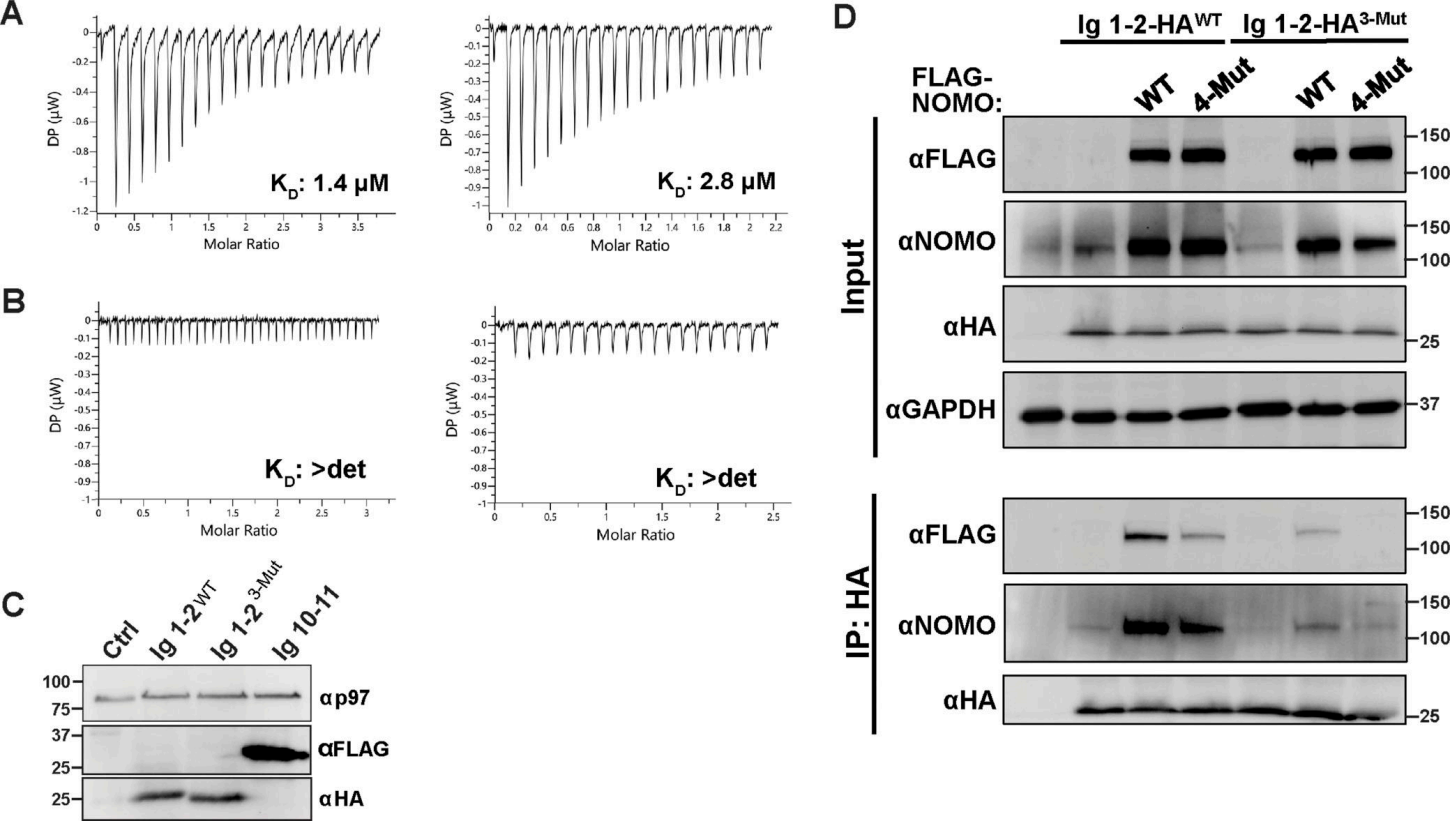
**ITC replicates and co-IP with WT and 3-Mut Ig 1–2. (A)** Isothermal titration calorimetry (ITC) replicates from Fig. 3 between WT Ig 1–2 and Ig 10–11. **(B)** ITC replicates for 3-Mut Ig 1–2 and Ig 10–11. Dissociation constants (K_D_) are indicated. *n* = 3 independent replicates were performed for each condition. **(C)** Immunoblot for expression of WT Ig 1–2-HA, 3-Mut Ig 1–2-HA, and Ig 10–11-FLAG in U2OS cells used for quantification in Fig. 3 G. **(D)** Co-immunoprecipitation (co-IP) analysis of interactions between HA-tagged Ig 1–2 constructs and WT or 4-Mut FL NOMO. Whole-cell lysates (input) and HA immunoprecipitates (IP: HA) were analyzed by immunoblot using antibodies against FLAG, NOMO, and HA. GAPDH serves as a loading control for input samples. Unprocessed blots are available in source data. Source data are available for this figure: SourceData FS3.

The inability of NOMO^4-Mut^ to rescue ER morphology under NOMO depletion prompted us to validate the Ig 1 and Ig 10–11 interaction with purified components. To this end, we individually expressed and purified recombinant His_6_-Ig 1–2 and His_6_-Ig 10–11 for in vitro–binding assays (Fig. 3 A). Due to the presence of a central beta sheet spanning Ig 1 and Ig 2, we purified a tandem construct to avoid structural perturbations. AlphaFold-Multimer generates a high-confidence model and interface between Ig 1–2 and Ig 10–11 (pTM = 0.85 and ipTM = 0.81, respectively) (Fig. S1, B and C). We turned to size-exclusion chromatography (SEC), incubating His_6_-Ig 1–2 with His_6_-Ig 10–11 for 1 h before loading the mixture onto a Superdex 75 pg column. Compared with the individual SEC elution profiles of His_6_-Ig 1–2 and His_6_-Ig 10–11, the mixture exhibited a shift to a higher molecular mass, consistent with the formation of a dimer (Fig. 3 B). Introducing three-point mutations at the salt bridge interface in Ig 1 (K66E, D70K, and D121K) (His_6_-Ig 1–2^3-Mut^) eliminated the observed shift (Fig. 3 C). To validate these results and quantify the interaction strength, we next performed isothermal calorimetry (ITC). Employing the same His_6_-Ig 1–2 and His_6_-Ig 10–11 fragments, we observed dimerization with a moderate affinity of 1.83 µM ± 0.84 (Fig. 3 D; and Fig. S3, A and B). In agreement with SEC results, we found no interaction between His_6_-Ig 1–2^3-Mut^ and His_6_-Ig 10–11 in ITC at the tested concentrations (Fig. 3 E, *n* = 3).

Considering that salt bridge interactions between Ig 1–2 and Ig 10–11 contribute to NOMO function (Fig. 2, D–F), we next investigated whether these fragments could interfere with endogenous NOMO activity. Toward this end, we individually expressed either Ig 1–2-HA or Ig 10–11-FLAG in WT U2OS cells and evaluated ER morphology by quantifying voids in transfected versus untransfected cells using calreticulin as an ER marker. Interestingly, expressing either construct induced a dominant-negative effect (Fig. 3, F and G; and Fig. S3 C) reminiscent of NOMO depletion, though to a lesser extent. However, expressing an Ig 1–2 construct harboring three-point mutations (Ig 1–2^3-Mut^) had a significantly reduced effect in inducing voids compared with the WT construct. To further investigate the Ig 1/10/11 interface, we performed a co-immunoprecipitation assay to assess the binding of WT Ig 1–2 and 3-Mut Ig 1–2 to endogenous and overexpressed NOMO. Our results demonstrate that WT Ig 1–2 interacts with overexpressed WT NOMO and, to a lesser extent, with 4-Mut NOMO. A similar trend was observed for endogenous NOMO. In contrast, 3-Mut Ig 1–2 exhibited minimal binding to WT NOMO and very little binding to 4-Mut NOMO (Fig. S3 D). This suggests that the NOMO assembly is dynamic, as ectopically expressed Ig modules can likely compete for the same interface to perturb NOMO function. Together, these results demonstrate that a moderate affinity interaction between Ig 1 and Ig 10–11 is critical for NOMO function.

### The Ig 1/10/11 interface contributes to a compact NOMO topology

**Figure 4.**
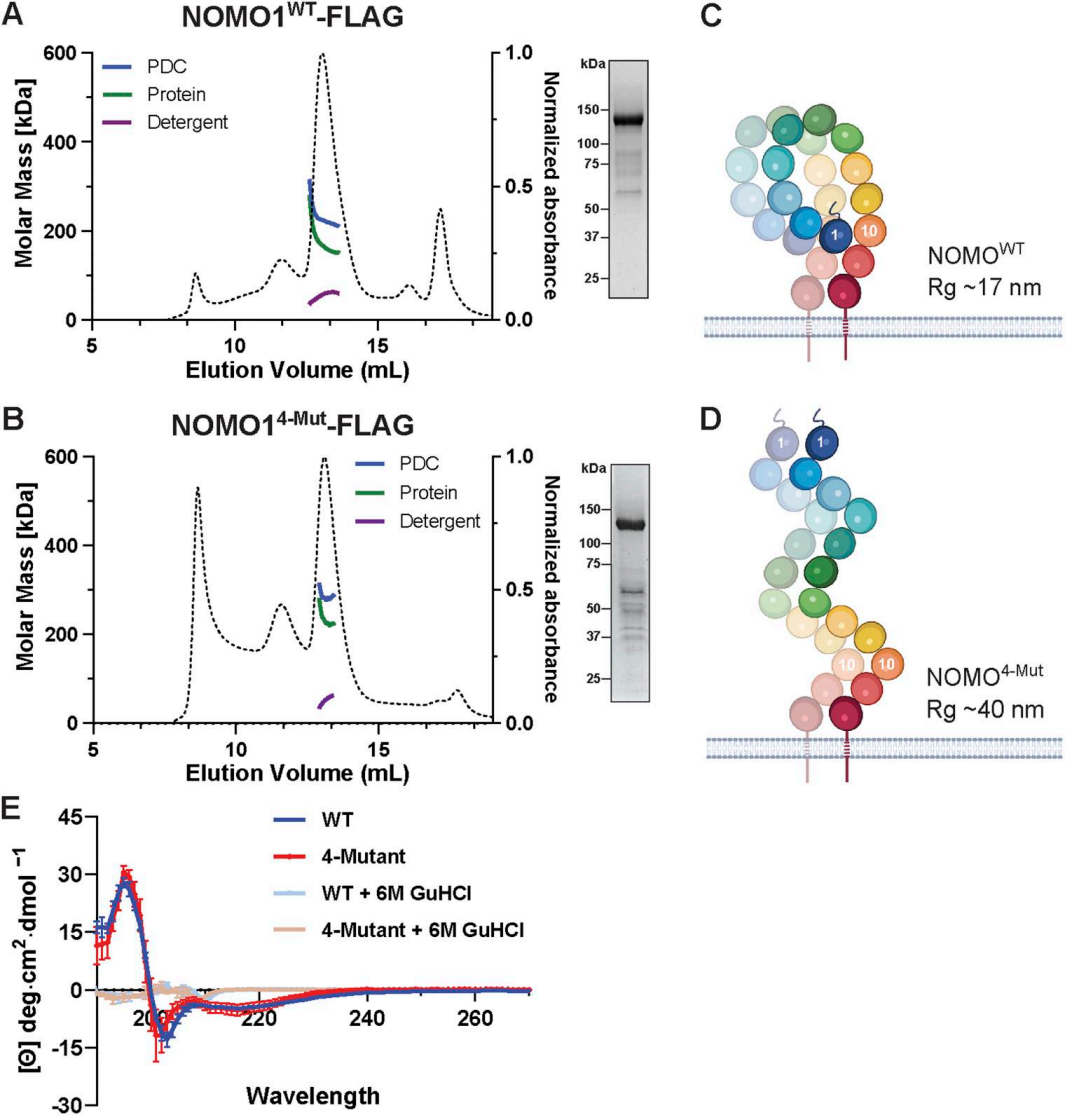
**NOMO architecture is shaped by the Ig 1/10/11 interface. (A)** SEC-MALS profile of FL WT NOMO (NOMO1^WT^-FLAG) on a Superose 6 column. The dashed line is the UV trace, molar mass represents the total molar mass of the PDC, and protein molar mass is the corrected molar mass to remove contribution from detergent. Inset, SDS-PAGE/Coomassie staining of the NOMO1^WT^-FLAG fraction obtained from preparative SEC. **(B)** SEC-MALS profile of the 4-mutant interface NOMO1 mutant, NOMO^4Mut^-FLAG, insert as in A. **(C and D)** Model representation drawn from the results shown in A and B. **(E)** Far-UV circular dichroism spectra of WT (blue) and 4-Mut (red) NOMO LD in the native conformation, and 6 M guanidine hydrochloride (GuHCl) treatment to induce unfolding (WT, tan; 4-mutant, light blue), as indicated by the loss of secondary structure. Curves represent normalized mean ± SD of *n* = 3 replicates. SEC-MALS, SEC linked to multi-angle light scattering. PDC, protein detergent complex. Source data are available for this figure: SourceData F4.

To evaluate the folding of NOMOr^4-Mut^ and the contribution of the Ig 1/10/11 interface to NOMO topology, we employed SEC coupled with multi-angle light scattering. In our previous analysis, we found that WT NOMO-FLAG has a radius of gyration (Rg) of ∼15 nm and a protein detergent complex-corrected molar mass ranging from 230 to 270 kDa, indicative of dimer formation (Amaya et al., 2021). Consistent with this, we observed a molar mass ranging from 220 to 270 kDa and an Rg of 15 nm for WT NOMO-FLAG (Fig. 4 A). The experimental radius is similar to that predicted by AlphaFold modeling of WT NOMO, which projects distances of 12–18 nm between distal points of the ER LD in three-dimensional space (Fig. S1 D). NOMOr^4-Mut^-FLAG exhibited a monodisperse peak with a comparable molar mass range to that of WT NOMO, suggesting it folds correctly and retains dimerization capability. However, a significantly greater Rg of ∼40 nm was observed (Fig. 4 B). The 12 Ig domains modeled by AlphaFold average ∼3.5 nm per fold, or 42 nm cumulatively, which is compatible with an elongated conformation for the interface-deficient NOMO or a looping topology adopted by WT NOMO (Fig. 4, C and D). To test if the interface-disrupting mutations do result in protein misfolding, we analyzed WT and mutant constructs encompassing the entire LD via circular dichroism. The circular dichroism spectra obtained for WT-LD and 4 mutant-LD were nearly identical, and highly sensitive to the chemical denaturant guanidium hydrochloride that was used as control (Fig. 4 E). These observations argue against major structural defects resulting from the interface mutations, although we cannot exclude the formal possibility that, in situ, additional cellular factors influence its conformation or stability.

### NOMO experiences interface-dependent force in situ

**Figure 5.**
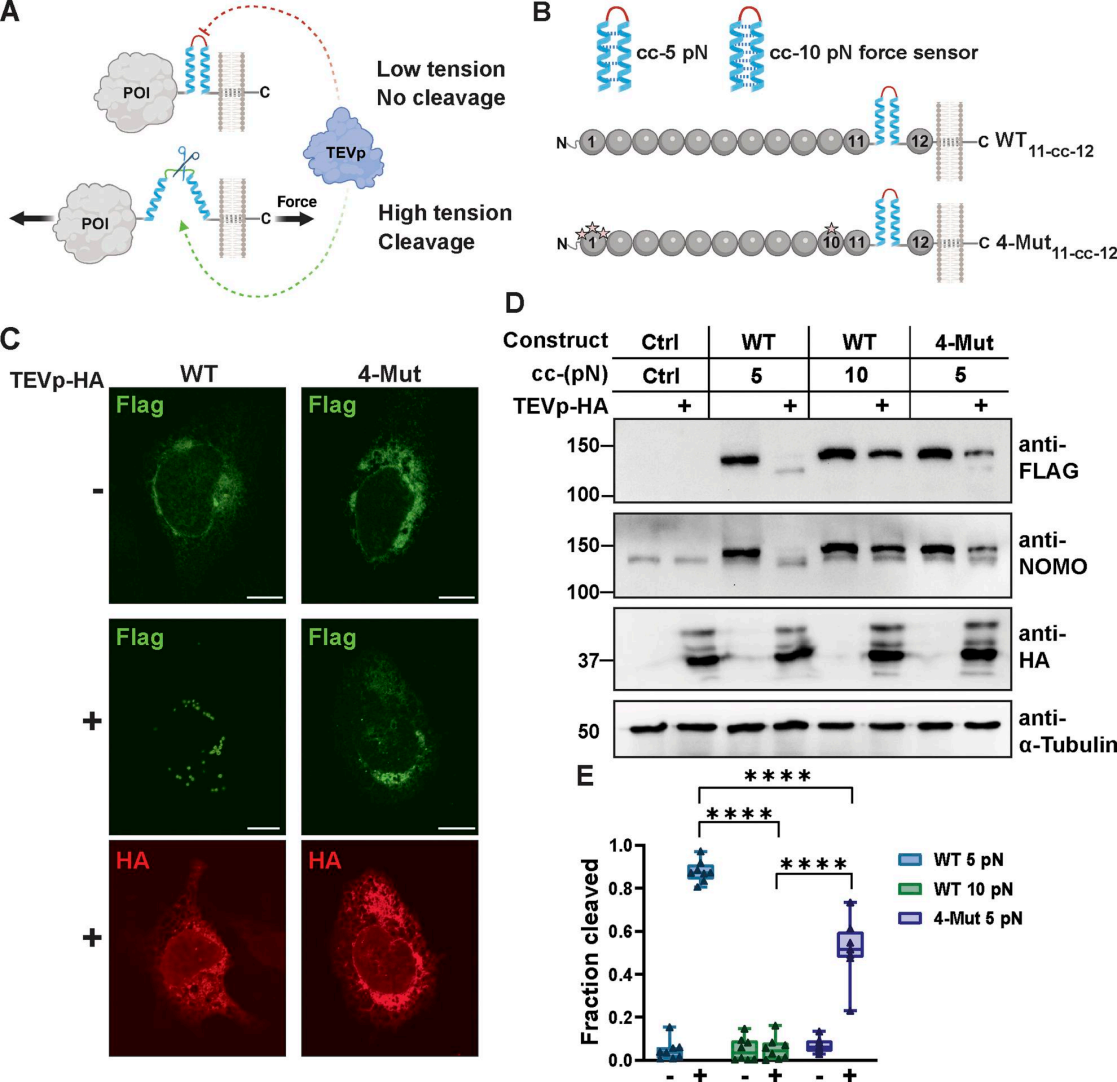
**NOMO is under single pN forces partly dependent on the Ig 1/10/11 interface. (A)** Illustration of the TEV cleavage-based readout. **(B)** Representation of the 5 pN and 10 pN coiled-coil (cc) sensors and their location between Igs 11 and 12 in WT and 4-mutant NOMO. **(C)** Immunofluorescence images of WT or 4-Mut FLAG-NOMO in either the absence (−) or presence (+) of TEVp. **(D)** Representative immunoblot of indicated cc-force sensors inserted in WT and 4-mutant NOMO when TEVp is absent or present (+). **(E)** Quantification of fraction of the indicated FLAG-NOMO construct cleaved under the absence (−) and presence (+) of TEVp. *N* = 8. Each condition was measured from *n* = 6 biological replicates. Statistical analyses were performed using an ordinary one-way ANOVA test; ****P < 0.0001. Source numerical data, immunoblot replicates, and unprocessed blots are available in source data. Source data are available for this figure: SourceData F5.

NOMO bears structural similarity to titin and several bacterial pili proteins with mechanosensitive Ig folds, some of which are known to unfold and refold in response to stress. These include BcpA, the major pilin subunit of *Bacillus cereus* (PDB ID 3KPT) (Budzik et al., 2009); RrgA and RrgC, pilus subunits of *Streptococcus pneumoniae* (PDB ID 2WW8 and 4OQ1) (Shaik et al., 2014; Echelman et al., 2016); and the minor pilin of *Streptococcus agalactiae* (PDB ID 3PHS) (Krishnan et al., 2007). Considering this, we sought to determine whether NOMO experiences force in the natural context of the ER. To do so, we implemented a cleavage-based readout that relies on TEV protease (TEVp) activity. This approach relies on the insertion of a TEV recognition sequence into a linker region between a coiled-coil interface of known mechanical strength (Ren et al., 2023). In this constrained conformation, the TEV recognition sequence is too distorted to be cleavable by the protease unless the coiled-coil interface is disrupted by a defined force threshold, which exposes the TEV recognition sequence for proteolysis (Fig. 5 A). The sensors were calibrated by optical tweezers that determined either 5 or 10 pN of force is required to unfold the coiled-coil and expose the cleavage site acted upon by TEVp co-transfected in cells (Ren et al., 2025, *Preprint*). We placed either sensor between Ig 11 and Ig 12 (NOMO^WT^_11-cc-12_) and appended an N-terminal signal sequence and C-terminal KDEL sequence to TEVp (TEVp-HA) to direct it to the ER lumen of WT U2OS cells in a genetic NOMO WT background (Fig. 5, B and C). Strikingly, the coiled-coil 5 pN sensor was cleaved completely, while the coiled-coil 10 pN sensor remained uncut (Fig. 5, D and E), indicating that NOMO is under force in the ER in the single pN range. We next asked if the previously defined Ig 1/10/11 interface is involved in force bearing. Installation of the 5 pN sensor into the interface mutant derivative (NOMO^4-Mut^_cc-5pN_) resulted in a 44% reduction in cleavage (Fig. 5, D and E), indicating decreased but not abolished force transmission. This suggests that the mechanical force experienced by NOMO is largely dependent on Ig 1/10/11, though potential contributions of differential protein stability or other cellular interactions cannot be fully excluded.

**Figure 6.**
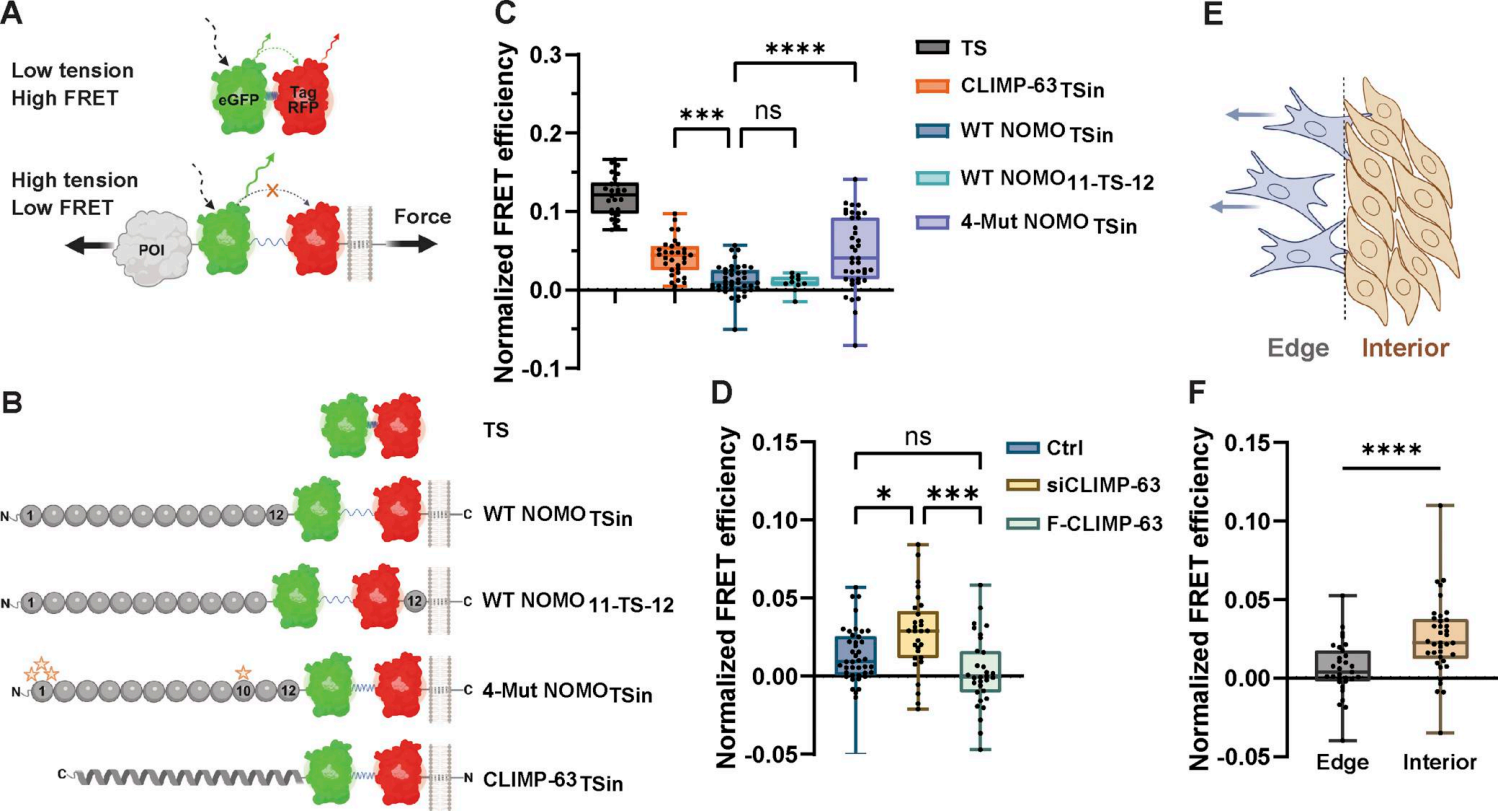
**Force on NOMO is modulated by ER sheet spacing and cell migration. (A)** Depiction of the FRET-based force sensor. **(B)** Rendering of the FRET sensor inserted within (TS_in_) constructs. Spheres depict Ig domains of NOMO, while a helix represents the coiled-coil topology of CLIMP-63. **(C)** Plots of normalized mean FRET efficiency ± SD for a TS only control, CLIMP-63 with TS inserted in the LD prior to the TM domain (CLIMP-63-TS_in_), WT NOMO with TS inserted in the LD prior to the TM domain (NOMO-TS_in_) or between Ig 11-12 (NOMO-TS_11-TS-12_), and 4-mutant NOMO (NOMO^4-Mut^-TSin). **(D)** Normalized mean FRET efficiency for NOMO-TS_in_ as in C or with CLIMP-63 silenced (siCLIMP-63) or overexpressed (F-CLIMP-63). NOMO-TS_in_ data are the same in C and D. **(E)** Schematic representation of the MEF3T3 wound migration assay. Cells at the wound edge extend protrusions and migrate into the gap, while interior confluent cells remain distal to the wound site. Arrows indicate the direction of migration. **(F)** NOMO-TS_in_ in MEF3T3 cells either migrating into a wound (Edge) or in a confluent area away from the wound (Internal). For all plots, errors reflect SD; *n* ≥ 30 cells for each condition measured from *n* ≥ 3 experiments. Statistical analyses were performed using a two-tailed unpaired Mann–Whitney test (F) or ordinary one-way ANOVA (C and D); ****P < 0.0001, ***P < 0.001, *P < 0.05, and ns, not significant. Source numerical data are available in source data.

As an orthogonal and complementary approach, we employed a well-established FRET-based TS (Kumar et al., 2016) that relies on an elastic flagelliform sensitive in the range of 1–6 pN (Grashoff et al., 2010). In its relaxed state, the nanospring is compact, resulting in high FRET; applied tension stretches the spring, reducing FRET intensity (Fig. 6 A). The module was inserted after Ig 12 and before the TM domain in either WT Flag-NOMO1 (NOMO-TS_in_), between Ig 11 and Ig 12 (NOMO_11-TS-12_), or 4-mutant Flag-NOMO1 (NOMO^4-Mut^-TS_in_) (Fig. 6 B). Consistent with the cleavage-based approach, WT NOMO was under high force (low FRET), while NOMO^4-Mut^ was under reduced force (moderate FRET) (Fig. 6 C). We additionally installed the TS in the ER spacer CLIMP-63 proximal to the ER luminal membrane and found it experiences less force than NOMO. To test whether mechanical strain through NOMO could be modulated, we used CLIMP-63 to vary ER luminal width, as we previously observed genetic interactions between CLIMP-63 and NOMO in modulating ER morphology (Amaya et al., 2021). Overexpression of CLIMP-63 leads to an increase in both the abundance and luminal width of sheets (Shibata et al., 2010), which moderately elevated tension through NOMO without reaching statistical significance (Fig. 6 D), potentially due to the sensor approaching the detection limit of ∼6 pN. Conversely, depletion of CLIMP-63 narrows the luminal width of the ER (Shen et al., 2019), which resulted in significantly reduced force on NOMO. Building on these findings, we next turned to a MEF3T3 wound migration assay, whereby we assessed the force dynamics on peripheral (edge) cells at the closing wound edge versus internal confluent cells. Analogous to the differential effects of focal adhesion engagement at peripheral versus interior keratinocyte colonies on the linker of nucleoskeleton and cytoskeleton complex (Carley et al., 2021), we hypothesized that cells at the edge experiencing higher mechanical strain (Sheetz et al., 1998; Merta et al., 2025) could transmit force to NOMO within the ER, while interior cells, with less engagement with the substrate, might propagate less force. Consistent with this, in edge cells migrating toward the wound, NOMO-TS_in_ experienced increased force, whereas in the interior cell population, tension on NOMO was significantly reduced (Fig. 6, E and F). Thus, we employed two independent readouts to arrive at the same conclusion: NOMO experiences force in the single pN range that responds to increased force experienced by cells, with a critical contribution from the Ig 1/10/11 interface.

### NOMO is required and relies on the Ig 1/10/11 interface for myogenesis in a C2C12 model, and depletion reduces nematode motility

**Figure S4.**
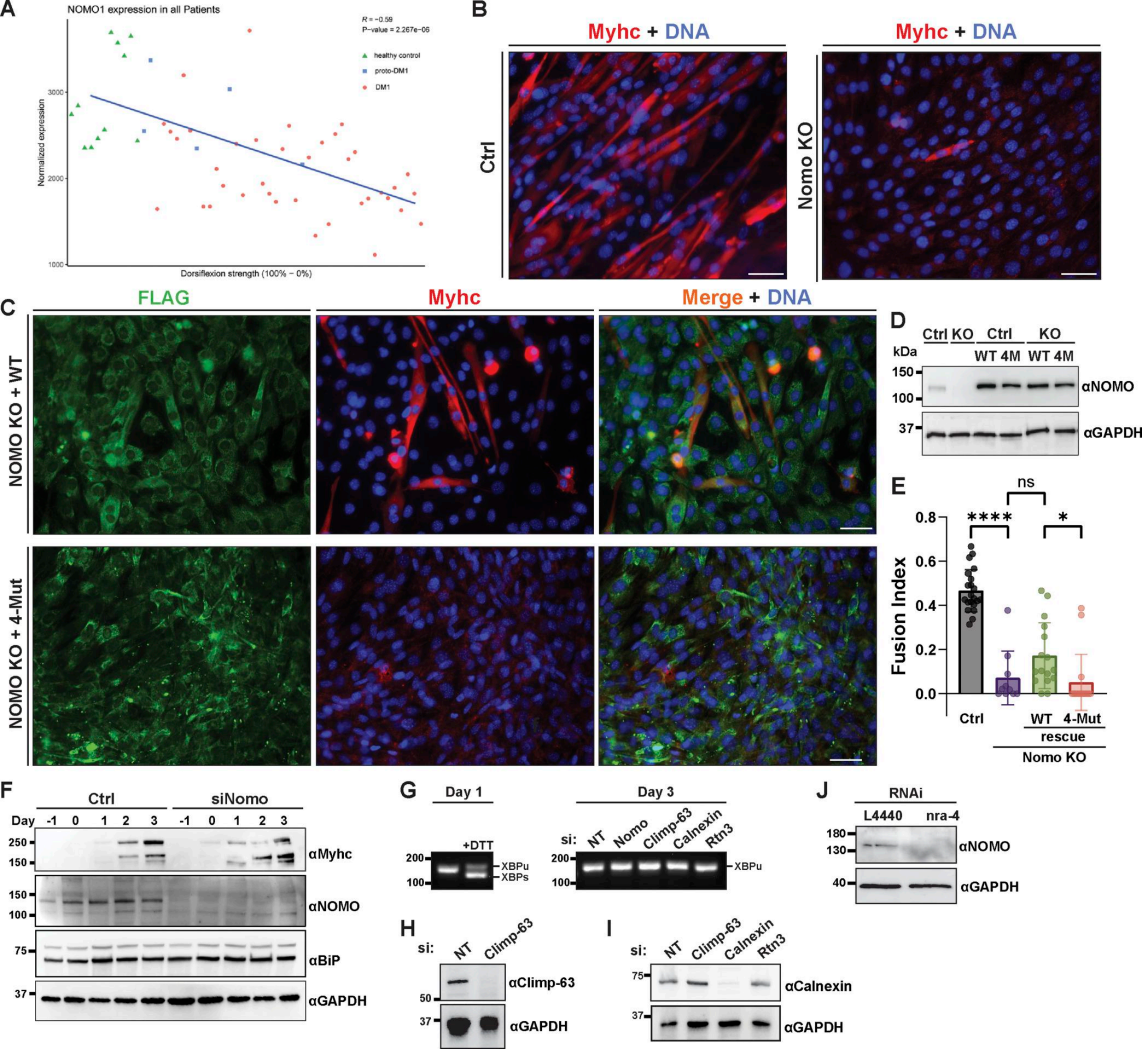
**Normalized expression of NOMO1 in** **tibialis** **anterior muscle biopsies and C2C12 differentiation in NOMO knockout cells. (A)** Dorsiflexion strength was measured in control and DM1 patients against NOMO transcript levels (R = −0.59). proto-DM1, an intermediate form of DM1 corresponding to fewer CTG repeats (50–100) than DM1 (>100) or healthy controls (<30). **(B)** Myogenesis was performed as in Fig. 6 A and immunostained for Myhc and stained for DNA with DAPI. **(C)** Immunoblot of control or NOMO-depleted (siNomo) myoblasts during indicated days of differentiation. **(D)** Assay performed as in B, with myoblasts stably expressing either WT (F-NOMO^WT^) or 4-Mut (F-NOMO^4-Mut^) constructs in Nomo knockout lines. **(E)** Quantification of myogenesis under indicated conditions, scored by fusion index. *n* = 21 frames, 2,433 nuclei (Ctrl), *n* = 10 frames, 1,411 nuclei (Nomo KO), *n* = 16 frames, 1,207 nuclei (Nomo KO, WT rescue), and *n* = 16 frames, 1,717 nuclei (Nomo KO, 4-Mut rescue). **(F)** Immunoblot comparing protein levels in control (Ctrl) and Nomo knockdown (siNomo) conditions during differentiation. Samples were collected at multiple time points up to day 3 of differentiation. Immunoblot analysis of myogenic markers in control and siNomo-treated cells over 4 days. **(G)** cDNA obtained from the indicated treated myotubes during day 1 or day 3 of differentiation, amplified via PCR using XBP-1–specific primers, separated by agarose, and stained with SYBR Safe. **(H and I)** Immunoblot of siRNA knockdown efficiency for Climp-63 and calnexin. GAPDH serves as a loading control. **(J)** Immunoblot analysis of nra-4 in Ctrl (L4440) and knockdown conditions and probed with anti-NOMO antibody. Statistical analyses were performed using an ordinary one-way ANOVA test; ****P < 0.0001, *P < 0.05, and ns, not significant. Error bars show min. to max. point range. All scale bars, 50 μm. Source numerical data and unprocessed blots are available in source data. Source data are available for this figure: SourceData FS4.

**Figure 7.**
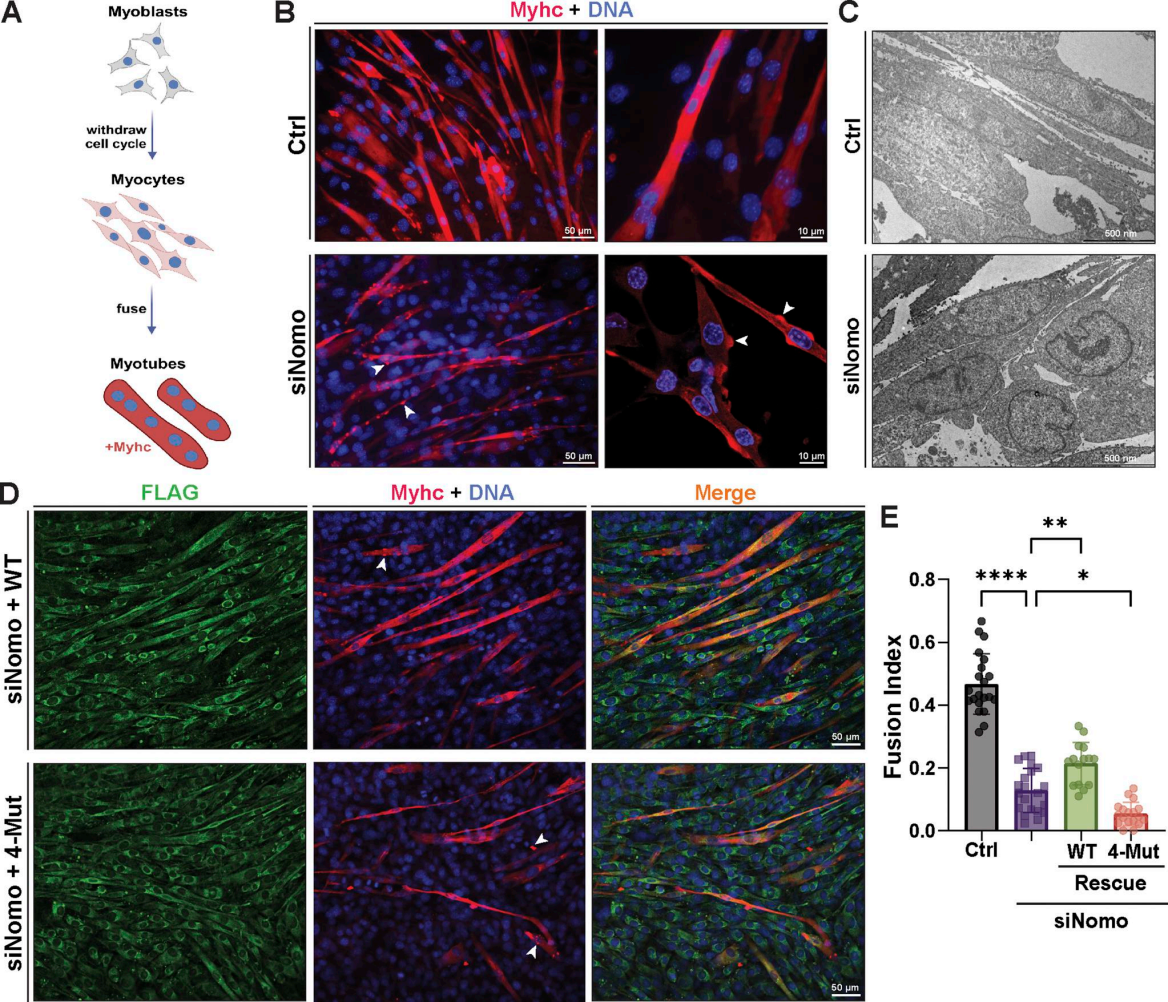
**NOMO is required for myogenesis and depends on the Ig 1/10/11 interface. (A)** Cartoon representation of C2C12 myogenesis, whereby myoblasts exit the cell cycle to differentiate into myocytes, which then fuse to form multinucleated myotubes expressing Myhc. **(B)** Fixed myotubes stained for Myhc and DAPI (DNA) 3 days after differentiation induction; left scale bars, 50 μm; right scale bars, 10 μm. **(C)** Electron micrograph of cells treated with either non-targeting (Ctrl) or Nomo siRNA (siNomo) during myoblast differentiation. **(D)** Representative images of the differentiation assay performed as in A, with myoblasts stably expressing siRNA-resistant F-NOMOr (WT) or F-NOMOr^4-Mut^ (4-Mut) constructs under Nomo knockdown. **(E)** Myogenesis quantification of conditions shown in B and C, scored by the mean fraction of 3+ nuclei in Myhc-expressing myotubes (fusion index). *N* = 21 frames, 2,788 nuclei (Ctrl), *N* = 21 frames, 2,677 nuclei (siNomo), *N* = 16 frames, 1,957 nuclei (siNomo, WT rescue), and *N* = 18 frames, 2,457 nuclei (siNomo, 4-Mut rescue) from *n* = 3 biological replicates. Statistical analyses were performed using an ordinary one-way ANOVA test; ****P < 0.0001, **P < 0.01, and *P < 0.05. Source numerical data are available in source data.

**Figure 8.**
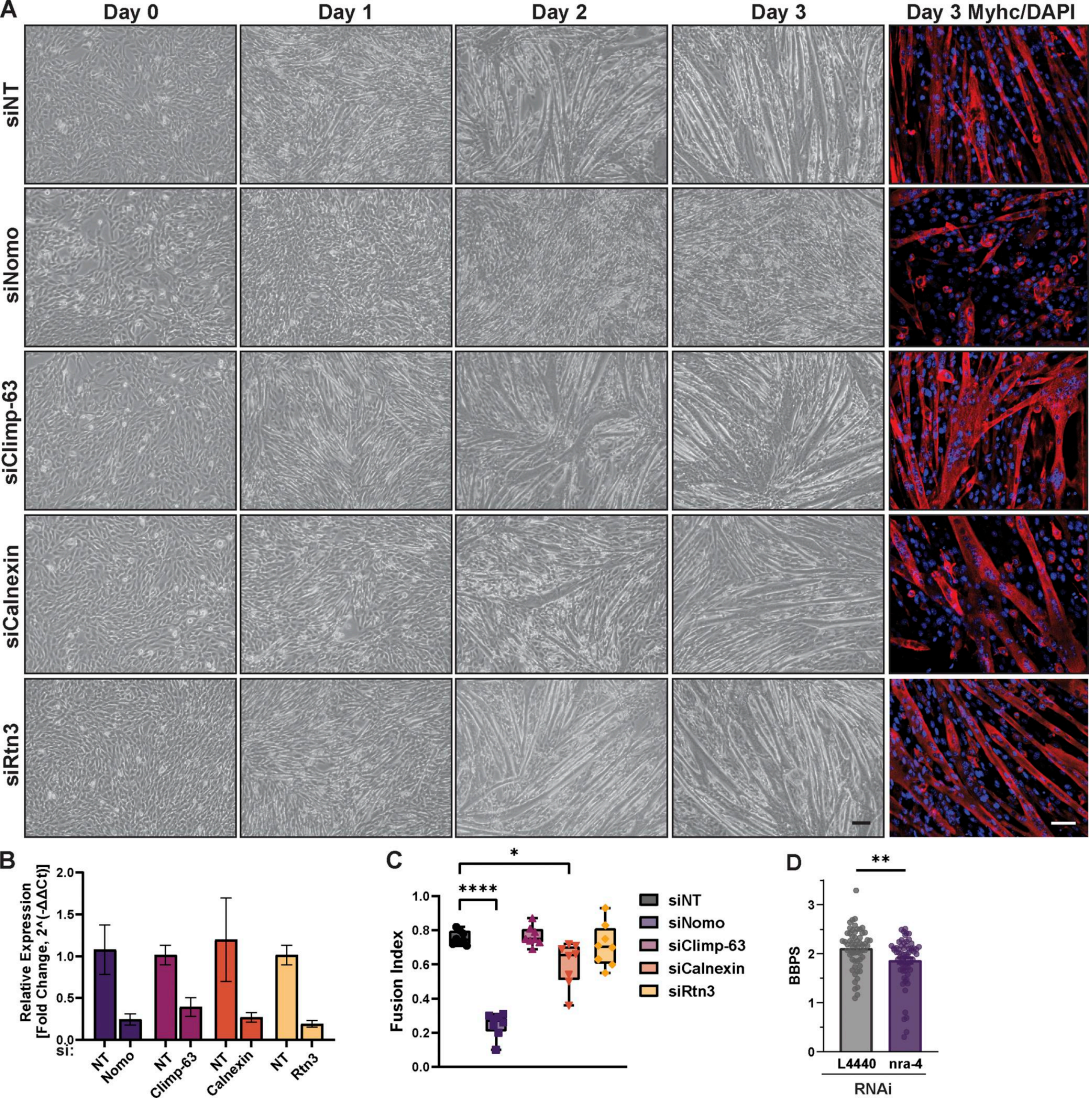
**Myogenesis defect is specific to NOMO rather than general ER dysfunction. (A)** Bright-field images showing myogenic differentiation over 4 days (day −3 to day 3) following siRNA-mediated knockdown of Nomo, Climp-63, calnexin, or Rtn3, with non-targeting (NT) control. Immunofluorescence images on day 3 show myotubes with Myhc (red) and DAPI (blue). Scale bar: 50 µm. **(B)** qPCR validation of knockdowns from A. **(C)** Quantification of the mean fusion index ± SD, representing the proportion of 2+ nuclei within Myhc-positive myotubes. *N* = 1,441 nuclei (siNT), *N* = 1,450 nuclei (siNomo), *N* = 1,653 nuclei (siClimp-63), *N* = 1,658 nuclei (siCalnexin), and *N* = 1,888 nuclei (siRtn3). Each condition was measured from *n* = 3 biological replicates. **(D)** Motility analysis measured by body bends per second (BBPS) of C*. elegans* challenged with either non-targeting (L4440) (*n* = 58) or nra-4–directed RNAi (*n* = 64). Data show mean ± SD from *n* = 3 biological replicates. Statistical analyses were performed using one-way ANOVA (C) or a two-tailed unpaired Mann–Whitney (D); ****P < 0.0001, **P < 0.01, and *P < 0.05. Source numerical data are available in source data.

**Figure S5.**
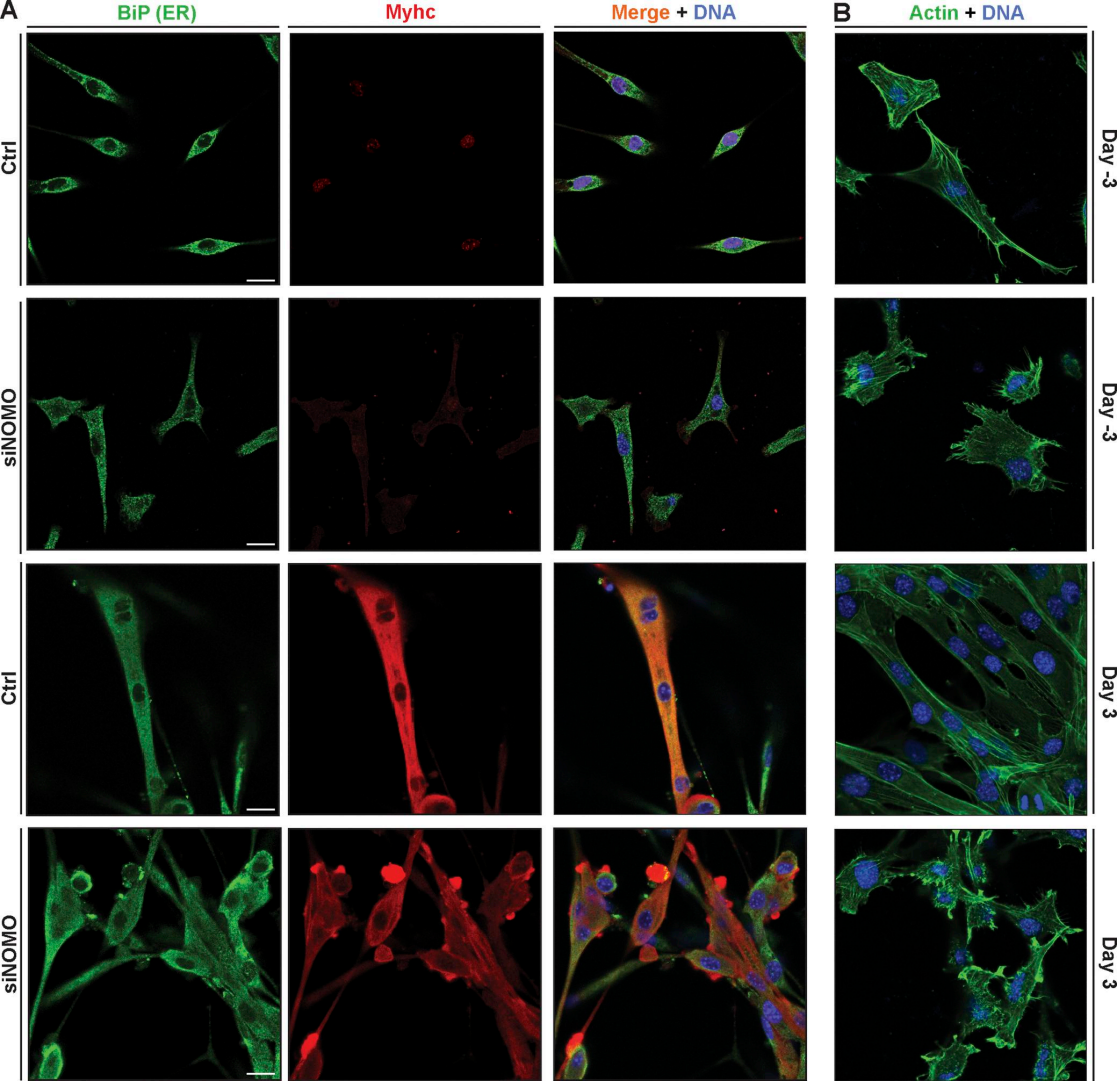
**ER, actin, and Myhc morphology effects under NOMO knockdown. (A)** Myoblast cells 3 days before differentiation (day −3) and after differentiation (day 3), immunostained for Myhc and ER marker BiP. **(B)** Conditions as in A, probed for actin by FITC-phalloidin.

Considering connections between NOMO and cardiogenesis (Zhang et al., 2015; Zhang et al., 2006; Xing et al., 2021), high expression in heart and muscle tissue (Uhlen et al., 2015), as well as load-bearing capabilities described above, we examined available transcriptomic data on muscular dystrophies. Interestingly, we identified differential NOMO1 expression in myotonic dystrophy type 1 (DM1) patients, with a positive correlation to dorsiflexion strength in the tibialis anterior muscle (R = −0.59) (Fig. S4 A) (Wang et al., 2019). These findings prompted us to investigate whether NOMO plays a role in myogenesis. Using the well-established mouse C2C12 in vitro differentiation model (Fig. 7 A), we performed RNA silencing experiments (Fig. 7, B and C), in addition to generating Nomo KO myoblast lines (Fig. S4, B–D). Nomo depletion minimally impacted myoblast survival and moderately delayed proliferation, arguing against nonspecific defects affecting viability. In differentiating myoblasts, cells fuse into multinucleated myotubes and express the differentiation marker type I myosin heavy chain (Myhc) (Fig. 7, A and B), among other protein indicators (Chal and Pourquie, 2017). Upon inducing myogenesis, however, Nomo-depleted cells exhibited significantly delayed and incomplete differentiation (Fig. 7, B and C lower panels, and Fig. 8 A, second row). Despite expressing comparable levels of Myhc to WT cells (Fig. S4 F)_,_ Nomo knockdown resulted in irregular clusters of Myhc rather than a uniform distribution along the myotubes, similar to the phenotype reported upon depletion of the fusion-promoting protein myomaker (Millay et al., 2013). Moreover, these cells formed thinner tubules containing fewer nuclei (Fig. 7, B, D, and E). In Nomo KO lines, failure to express Myhc may be attributable in part to repeated passaging to isolate clones, likely exacerbating myogenesis impairment by NOMO depletion (Fig. S4, B–E) (VanGenderen et al., 2022). However, Nomo depletion via siRNA knockdown is highly efficient and can be used as an alternative to avoid prolonged passaging (Fig. S4 F). We additionally probed for effects to ER morphology and actin organization in myoblast lines lacking Nomo (Fig. S5, A and B). Upon silencing Nomo, ER morphology was moderately perturbed in undifferentiated myoblasts, while actin organization was irregular in both undifferentiated (day −3) and differentiating myotubes (day 3). The pervasive void phenotype observed in other cell lines was not penetrant in the myoblasts, either before or after differentiation (Fig. S5 A). Overall, Nomo depletion led to disrupted and mislocalized Myhc expression (see arrowheads, Fig. 7, B–D), ultimately resulting in failure to establish myotubes in Nomo KO and KD conditions (Fig. 7, B–E; and Fig. S4, B, D, and E). This was also evident in electron micrographs, which revealed a nuclear envelope (NE) deformation phenotype upon NOMO depletion (Fig. 7 C).

To verify the specificity of Nomo depletion, we conducted Nomo rescue experiments in Nomo KD and KO backgrounds. C2C12 lines were engineered to stably express WT human F-NOMOr at moderate levels, as confirmed by IF and immunoblot (Fig. 7 D; and Fig. S4, C and D). In Nomo-silenced samples, expression of human WT NOMO (resistant to siRNA-targeting murine Nomo) significantly enhanced differentiation, although it remained below control conditions (Fig. 7, D and E). We attribute this incomplete rescue to the use of a human construct in a mouse line, which may affect additional interactions beyond homotypic ones, as well as the prolonged culturing required to generate stable cell lines that may limit the differentiation potential of myocytes. Next, we sought to determine whether the Ig 1/10/11 interface critical for ER morphology and force-bearing is necessary for myogenesis. To this end, we produced stably expressing F-NOMOr^4-Mut^ in WT and Nomo KO myoblast lines. Upon inducing differentiation under Nomo knockdown, 4-Mut–expressing cells exhibited more severe delays in myotube formation and Myhc patterning than Nomo-silenced conditions (Fig. 7, D and E).

To understand whether a general ER defect underlies the myogenesis defect, we also performed knockdown experiments on the ER-resident proteins Climp-63 and Rtn3, chosen as a major players in the context of ER morphology (Shibata et al., 2009), as well as calnexin, a major player in ER protein quality control (Kozlov and Gehring, 2020). Silencing Nomo significantly disrupted myogenesis—as evidenced by a pronounced reduction in the fusion index—whereas depletion of the other proteins did not lead to similar impairments (Fig. 8, A–C and Fig. S4, G–I). In addition, we failed to observe discernible differences in X-box binding protein 1 (XBP-1) splicing or UPR target BiP expression upon NOMO depletion (Fig. S4, F and G), suggesting that the observed differentiation defects are unlikely to be caused by severe ER stress or result from nonspecific morphological changes of the ER. Together, these results support the conclusion that NOMO is specifically required for myogenesis.

If NOMO is indeed required for muscle function, locomotion defects would be expected on an organismal level. To test this idea in a metazoan while avoiding viability issues arising from potential heart defects, we employed a nematode model (nematodes lack a heart). We directly compared the motility of *C. elegans* challenged with control RNAi with those in which the direct NOMO homolog *nra-4* was targeted via RNAi (Fig. 8 D and Fig. S4 J). The motility of nematodes challenged with *nra-4*–directed RNAi was significantly compromised relative to control animals (Fig. 8 D, Videos 1, and 2). This result supports our functional assignment of NOMO to a critical role in muscle function.

**Video 1.** **The motility of *C. elegans* expressing *myo-3p::myo-3::gfp* was assessed on day 4 of adulthood following two generations of control RNAi (L4440) via feeding.** Nematodes were synchronized by egg laying and recorded in M9 media after 30-s acclimation. Videos were acquired at 7.8× magnification and 25 fps using a Leica M205FA stereo microscope. Motility, measured as body bends per second (BBPS), was quantified using WormLab software (Fig. 8 D).

**Video 2.** **The motility of *C. elegans* expressing *myo-3p::myo-3::gfp* was evaluated on day 4 of adulthood after two generations of RNAi-targeting nra-4 through feeding.** Nematodes were synchronized by egg laying and recorded in M9 media following a 30-s acclimation. Videos were captured at 7.8× magnification and 25 fps using a Leica M205FA stereo microscope. Motility (BBPS) was analyzed using WormLab software (Fig. 8 D).

## Discussion

This study establishes NOMO as a mechanosensitive protein essential for myogenesis and identifies key molecular features that are critical for modulating tension and functionality. Our findings not only highlight NOMO’s involvement in muscle differentiation but also advance our understanding of how intracellular organelles and proteins process mechanical stimuli.

ER morphology is vital to cellular function and depends on a suite of protein shapers and spacers (Westrate et al., 2015; Shibata et al., 2010). We previously demonstrated that NOMO-depleted cells undergo substantial ER rearrangement (Amaya et al., 2021). The restoration of this phenotype by expression of NOMO or ER-shaping proteins atlastin-2 or CLIMP-63, together with evidence that NOMO directly affects sheet spacing, established its role in maintaining ER structure. Despite these findings, the precise mechanisms by which NOMO, with its tandem Ig folds resembling force-bearing proteins, preserves ER morphology remained unclear. Here, we find that a highly conserved interface between Ig 1 and Ig 10–11 is central to NOMO function, as mutations disrupting these interactions severely compromise NOMO’s ability to rescue ER morphology, buffer mechanical forces, and promote myogenesis (Fig. 2, D and F; Fig. 5 C; Fig. 6 C; and Fig. 7, C and D). Our analyses furthermore demonstrate that interactions between Ig 1 and Ig 10/11 are of modest (∼1 µM) affinity (Fig. 3 D) yet are essential for the metastable positioning of NOMO within the ER and clearly contribute to the comparatively slow diffusional mobility (Fig. 2, E–G and Fig. 4).

**Figure 9.**
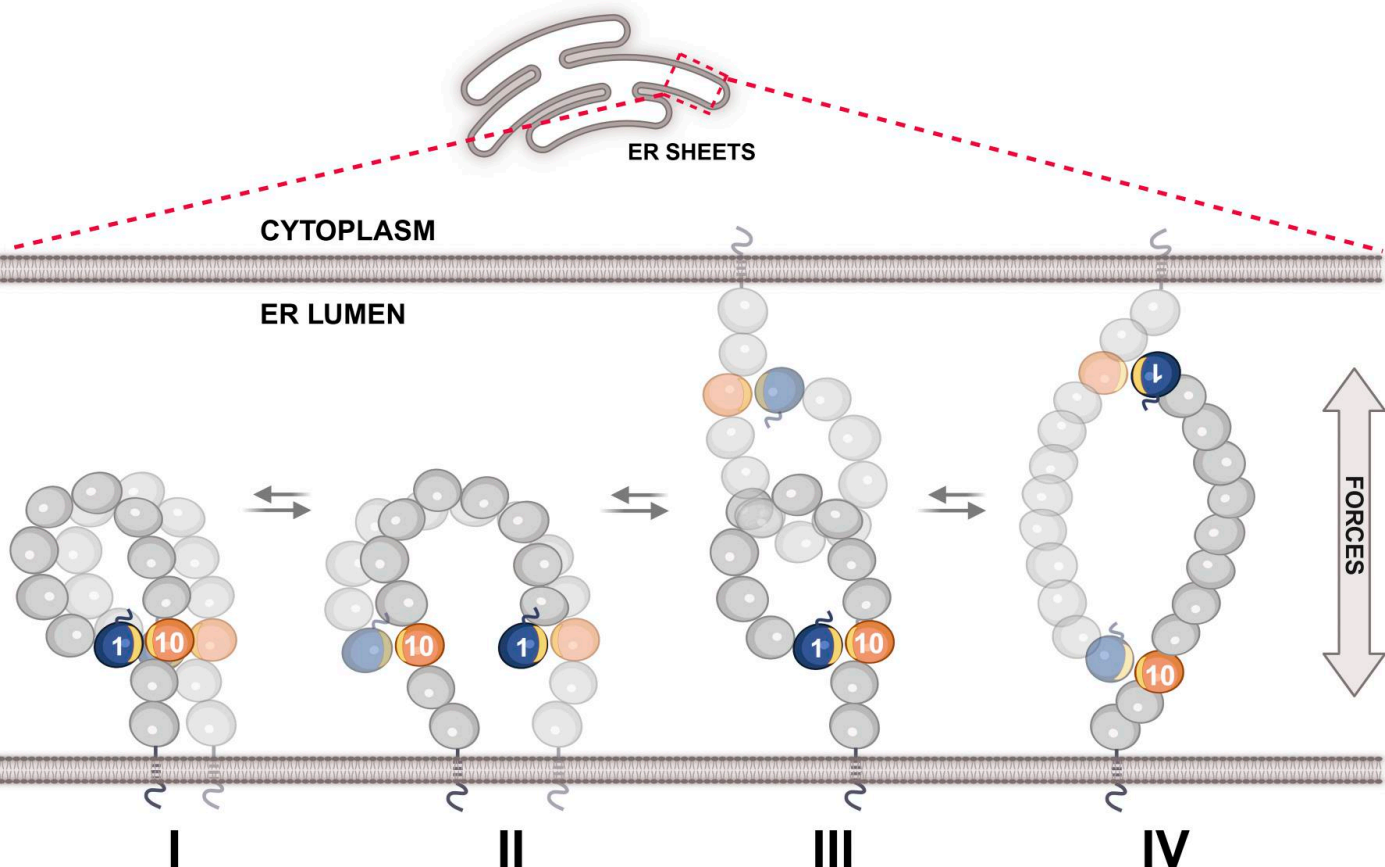
**Models of NOMO assembly in the ER.** NOMO dimerization is represented in cis in models I and II and in trans in models III and IV. These models serve as the basic building blocks of NOMO oligomerization. The Ig 1/10/11 interface (yellow sliver) is shown intramolecularly in I and III and intermolecularly in II and IV. Ig domains are represented as circles, with Ig 1 and Ig 10 colored blue and orange, respectively.

These findings suggest that a delicate balance between stability and dynamic flexibility may be linked to the oligomerization of NOMO through Ig domains 1, 10, and 11. We envision that NOMO may assume dynamically interchangeable and coexisting assemblies (Fig. 9) within the ER lumen, derived from the basic unit of the NOMO dimer (Fig. 4) and capable of higher-order homomeric assemblies, as well as potential interactions with other ER components. The identical interfaces in NOMO assemblies would allow for energetically “neutral” switching among conformations. Lower NOMO concentrations likely favor a looping model (Fig. 1 A and Fig. 9, models I and III), while higher densities promote intermolecular binding (Fig. 9, models II and IV). The ability of isolated WT Ig 1–2 or Ig 10–11 segments to induce a dominant-negative phenotype (Fig. 3, F and G) suggests a dynamic interconversion of these assembly modes. A flexible equilibrium would support bulk cargo transport through the ER, maintain membrane integrity, and enable dynamic adaptation of ER morphology, including the sliding of parallel membranes in response to mechanical challenges (Helle et al., 2013). Clearly, additional work is necessary to test these ideas in the future.

Apart from homotypic assemblies, we and others have proposed that NOMO serves additional functions, e.g., by restricting other membrane protein complexes to sheet regions of the ER (Amaya et al., 2021; Lewis et al., 2024). NOMO is an integral component of the back of Sec61 complex, where it may anchor nicalin and TMEM147 to regions of sheets where protein synthesis occurs. While not depicted, the modeled states would accommodate a NOMO-nicalin interface at the backside of Ig 11–12 (Page et al., 2023, *Preprint*; Gemmer et al., 2024).

The dynamic states of NOMO may be influenced by forces acting on the ER, which are important for maintaining cellular function (Feng and Kornmann, 2018; Goyal and Blackstone, 2013; Song et al., 2024). Mechanical stimuli are transmitted through cytoskeletal interactions, membrane-associated proteins, and interactions with other organelles (McGinn et al., 2021), which initiate pathways that control cellular functions such as gene and protein expression, organelle dynamics, and cell fate decisions (Dupont and Wickstrom, 2022; Orr et al., 2006; Romani et al., 2021). Tension-sensitive probes employed in this study demonstrate that NOMO experiences mechanical stress in the range of ∼5 pN (Figs. 5 and 6), similar to that documented in other force-responsive proteins (Kumar et al., 2016; Carley et al., 2021). Sheet spacing via CLIMP-63, modulation of forces during cell migration, and perturbation of the Ig 1/10/11 interface via mutations modulate the intensity of strain experienced by NOMO (Fig. 6, C–F), emphasizing the importance of environment and conformation in mechanosensitivity (Lombardi and Lammerding, 2011). In the future, it will be interesting to test if NOMO associates with cytoskeletal components via the cytosolic tail, as was reported for CLIMP-63 (Klopfenstein et al., 1998), and whether it cooperates with other ER or NE proteins to establish a force-responsive network. Notably, NOMO’s Ig domains closely resemble energy-absorbing regions in cadherins, titin, and pilus adhesive proteins (Yonemura et al., 2010; Echelman et al., 2016; Tskhovrebova et al., 1997; Ren and Berro, 2022), evocative of a dissipative architecture designed to handle mechanical strain. Additional work will be needed to understand how dynamic forces and interactions with ER components affect NOMO’s structure and whether the Ig domains can act as bona fide force buffers via reversible unfolding/refolding, akin to titin (Tskhovrebova et al., 1997). Similarly, the relation to the observed void phenotype warrants future exploration. The voids may result from a lack of intermembrane cohesion that would normally be mediated by NOMO dimerization across the ER lumen, consistent with (1) the inability of NOMO derivatives defective in cross-dimerization to complement this phenotype (Fig. 2, B and D) and (2) the partial rescue of this phenotype upon overexpression of the ER spacer Climp63 (Amaya et al., 2021). As an alternate but not mutually exclusive scenario, voids might be related to an ER-phagic response to counteract compromised ER integrity resulting from a failure to buffer mechanical strain, consistent with the previously observed upregulation of the autophagy marker LC3-II upon NOMO depletion (Amaya et al., 2021). Readouts to test these possibilities directly are being developed currently.

We found that depleting NOMO severely impedes C2C12 myogenesis (Fig. 7, B and E; Fig. 8, A and C; and Fig. S4 E), highlighting a crucial function for NOMO in promoting muscle cell differentiation. Interestingly, NOMO is structurally similar to gp210, a nuclear pore complex component also featuring tandem Ig folds and implicated in myocyte differentiation (Gomez-Cavazos and Hetzer, 2015). Given the pronounced nuclear abnormalities observed in NOMO-depleted myotubes (Fig. 7 C) and earlier findings highlighting essential roles for the ER and nucleus in muscle development and function (D’Angelo et al., 2012; Hao and Starr, 2019; Kalukula et al., 2022; Janota et al., 2020; Turishcheva et al., 2022), continued research is warranted to assign the precise mechanisms linking NOMO to myogenesis and scrutinize a possible functional interplay with gp210 and the linker of nucleoskeleton and cytoskeleton complex in supporting the mechanical properties of the NE (Sosa et al., 2013).

Findings from our study, along with those of others (Haffner et al., 2004), provide evidence for NOMO’s involvement in cellular differentiation processes and suggest broader implications in tissue development and homeostasis. Our identification of NOMO as a force-bearing protein involved in myoblast differentiation, DM1 presentation, and nematode motility established in this study establishes its critical role in striated muscle physiology, while also establishing that the ER lumen experiences significant forces. Future research will focus on elucidating NOMO’s mechanistic roles in relation to cellular forces and exploring its potential as a therapeutic target for muscular and cardiac disorders.

## Materials and methods

### Tissue culture

All cells were obtained from ATCC. U2OS, MEF3T3, and HeLa cells were cultured as previously described (Amaya et al., 2021; Prophet et al., 2022). Briefly, cells were cultured in DMEM supplemented with 10% (vol/vol) FBS (Thermo Fisher Scientific) and 100 U ml^−1^ of penicillin-streptomycin (Thermo Fisher Scientific).

### Immunofluorescence

Cells cultured on coverslips were washed once with PBS warmed to 37°C and then fixed in 4% paraformaldehyde (+0.1% glutaraldehyde for any samples analyzing ER structure) at room temperature for 15 min and washed twice with PBS. Samples were permeabilized with 0.3% (vol/vol) Triton X-100 for 3 min, washed three times with PBS for 5 min each, and blocked with 3% BSA in PBS for 1 h at room temperature. Coverslips were then placed in a humidity chamber and incubated with primary antibodies diluted in 3% BSA in PBS for 1 h at room temperature. Following three PBS washes for 5 min each, coverslips were once more placed in the humidity chamber and incubated with secondary antibodies in 3% (wt/vol) BSA in PBS for 1 h at room temperature in the dark. Cells were washed with PBS, incubated with Hoechst 33342 nuclear stain (Life Technologies) for 1 min, and washed twice in PBS for 5 min before being mounted onto slides using Fluoromount-G (Southern Biotech). Primary antibodies were diluted 1:500: anti-FLAG produced in mouse (F1804; Millipore Sigma), anti-FLAG produced in rabbit (D6W5B; Cell Signaling), anti-HA (11867423001; Roche), anti-calreticulin (27298-1-AP; Proteintech), anti-myosin hc (MAB4470; R&D Systems), and diluted 1:1,000 phalloidin FITC reagent (ab235137; Abcam). Secondary antibodies were conjugated to Alexa Fluor 488 (Life Technologies) or Alexa Fluor 568 (Life Technologies).

Images were acquired on an LSM 880 laser scanning confocal microscope (Zeiss) with Airyscan using a C Plan-Apochromat 63×/1.40 oil DIC M27 objective using ZEN 2.1 software (Zeiss). Standard wide-field images were obtained on a Zeiss Axio Observer D1 microscope with a 63× oil immersion objective and an AxioCam MRm microscope camera using AxioVision 4.8 software (Zeiss). For live-cell imaging, cells were grown in a µ-slide 8-well glass-bottom chamber (80827; ibidi) and imaged in a CO_2_-, temperature-, and humidity-controlled Tokai Hit Stage Top Incubator. Images were acquired on an inverted Nikon Ti microscope equipped with a Yokogawa CSU-X1 confocal scanner unit with solid-state 100-mW 488-nm and 50-mW 561-nm lasers, using a 60× 1.4 NA plan Apo oil immersion objective lens and a Hamamatsu ORCA R-2 Digital CCD Camera.

### Electron microscopy

Cells grown on 10-cm^2^ plates were fixed with 2.5% glutaraldehyde in 0.1 M sodium cacodylate buffer (pH 7.4) with 2 mM calcium chloride for 1 h. After, cells were rinsed and further fixed for 1 h in 1% osmium tetroxide and 1.5% potassium ferrocyanide. Samples were then placed in 1% thiocarbohydrazide for 30 min, rinsed, and incubated with 1% osmium tetroxide for 1 h, followed by a wash with distilled water. The samples underwent overnight en bloc staining in an aqueous solution of 0.25% uranyl acetate, followed by rinsing with distilled water. They were then dehydrated through a graded ethanol series ranging from 30 to 100%, followed by treatment with 100% propylene oxide. Over 2 days, the samples were infiltrated with Durcupan resin (Electron Microscopy Sciences), embedded in silicone molds, and cured at 60°C for a minimum of 48 h. The hardened blocks were sectioned using a Leica UltraCut UC7, and 60-nm sections were collected on formvar/carbon-coated grids. Imaging was performed on an FEI Tecnai Biotwin TEM at 80 kV, with images captured using an AMT NanoSprint15 MK2 sCMOS camera.

### Cloning, transient RNAi knockdowns, and transient transfections

Constructs were cloned by Gibson assembly as previously described (Amaya et al., 2021) from Dharmacon plasmids containing the NOMO1 gene in a pcDNA3.1^+^ vector. siRNA KD experiments were executed as previously described (Rampello et al., 2020). Briefly, cells were seeded in either a 12-well plate at 0.5 × 10^5^ cells/well or a 6-well plate at 1 × 10^5^ cells/well. For KD, lipofectamine RNAimax (Invitrogen) was used to transfect 50 nM of either non-targeting or NOMO1 siRNA (Horizon Discovery), followed by a second transfection at 24 h and harvesting or fixation at 72 h. For rescues, Lipofectamine 3000 (Invitrogen) was used to transfect the siRNA/DNA mixture. Each siRNA and plasmid combination was tested in four independent experiments. Following immunofluorescence (described above), CellProfiler software (Stirling et al., 2021) was used for void and myogenesis quantification (described in further detail below). For void quantification, areas lacking calreticulin staining with diameters between ∼0.5 and 5 µm were measured. The total area of holes per cell was then compared with the total area occupied by calreticulin ER staining and reported as a percentage.

### NOMO antibody production

Rabbit antiserum was produced against FL FLAG-tagged NOMO1 (Covance). The anti-NOMO antibody was further immunoaffinity purified using His_6_-Ig 10–11 coupled to nickel beads, followed by washing and elution of NOMO according to the established protocols (Harlow and Lane, 2006).

### Unfolded protein response induction

To induce unfolded protein response, 1 mM DTT was added to myotube cell cultures at day 1 of differentiation induction and incubated at 37°C for 3 h. Total RNA was extracted by RNeasy Plus mini kit (Qiagen). The cDNA of XBP-1 was amplified by RT-PCR using a first strand cDNA synthesis kit (Roche). XBP-1 splicing was analyzed by RT-PCR using primers 5′-AAC​ACG​CTT​GGG​AAT​GGA​CA-3′ and 5′-CCA​TGG​GAA​GAT​GTT​CTG​GGG-3′, flanking the region encompassing the unspliced and spliced XBP-1. The PCR fragments were separated by 3% agarose in Tris-acetate-EDTA buffer and visualized by SYBR Safe DNA gel stain.

### Lentivirus production and myoblast transduction

Generating lentivirus for stable integration of NOMO constructs into myoblast cell lines was adapted from previously described methods (Tsai et al., 2019; Gomez-Cavazos and Hetzer, 2015). Low-passage HEK293T cells were seeded in 10-cm plates at 3.5 × 10^6^ cells per plate. The following day, each plate containing 6 ml of medium was co-transfected with three plasmids: MMLV gag/pol (2 μg), viral envelope protein VSV-G (1 μg), and either pBABE-puro-F-NOMO1r (6 μg) or pBABE-puro-F-NOMO1r^4-Mut^ (6 μg) using 27 μl of X-tremeGENE 9 (Roche). Supernatants were filtered after 72 h through a 0.45-μm filter unit. To proliferate myoblast cells seeded in 6-well plates at 1 × 10^5^ cells/well (∼30% confluency) in 2 ml medium supplemented with 4 μg/ml of polybrene, virus was serially diluted from 50 to 500 μl. After 48 h, 2.5 μg/ml of puromycin was added to each well, including to a control well lacking transduction, and incubated for 48 h. Cells were seeded in 15-cm plates at low density (∼150 cells/plate), and isolated single clonal clusters were validated by immunofluorescence.

### Immunoblot analysis

For protein extracts, cells were lysed in 1% SDS buffer with benzonase and incubated at 95°C for 5 min. For analysis of C2C12 attached and detached populations, detached cells were harvested by collecting the floating and loosely attached cells in the media, and recovered living cells by scraping the plates. Protein concentration was determined using the bicinchoninic acid reagent (Thermo Fisher Scientific). For immunoblot blot analysis, equal amounts of protein (10–20 µg) were resolved by 8% SDS-PAGE gels and transferred onto polyvinylidene fluoride membranes (Bio-Rad). The membranes were blocked in 4% (wt/vol) nonfat milk in PBS + 0.1% (vol/vol) Tween-20 (Sigma-Aldrich). Primary and horseradish peroxidase–conjugated secondary antibodies were diluted in blocking buffer. The blots were visualized by chemiluminescence on a ChemiDoc gel imaging system (Bio-Rad). Band intensities were quantified using ImageJ (Fiji), and background was subtracted from each measurement. FLAG/NOMO levels were normalized to GAPDH levels in each lane to control for loading variation. Relative protein levels were compared between TEVp-present (+) and TEVp-absent (−) conditions.

### FRAP

FRAP experiments were performed using an LSM 880 laser scanning confocal microscope (Zeiss) using a C Plan-Apochromat 63×/1.40 oil DIC M27 objective using ZEN 2.1 software (Zeiss). Experiments were done at 37°C with 5% CO_2_ using a live-cell chamber system. For each acquisition, NOMO-TS_in_ was bleached using the 488-nm laser. The eGFP in TS was subjected to photobleaching, leaving tagRFP unperturbed. Between five pre-bleach images were acquired, and postbleach images were acquired every 0.15 s for 60 s. To monitor recovery, a circular ROI of 1 µm was used to measure the average fluorescence intensity before and after photobleaching. These values were normalized to the pre-bleach frame and graphed relative to bleaching. Data from at least three experiments totaling at least 50 cells for each construct were collected, and the averaged FRAP measurements were plotted in GraphPad Prism 10 and fit to a nonlinear regression exponential curve.

### FRET

The eGFP-TagRFP TS module, a kind gift from Dr. Martin Schwartz (Yale University, New Haven, CT, USA), was inserted after NOMO Ig 12 and prior to the transmembrane domain. Cells were plated on glass-bottomed dishes (CELLTREAT) coated with poly-L-lysine (P9155; Sigma-Aldrich) and transfected using Lipofectamine 2000 or Lipofectamine 3000 reagent (Thermo Fisher Scientific) according to the manufacturer’s instructions and imaged the following day. Untransfected cells were used as dark controls. Imaging was performed on an LSM 880 laser scanning confocal microscope (Zeiss) using a C Plan-Apochromat 63×/1.40 oil DIC M27 objective using ZEN 2.1 software (Zeiss) with a stage maintained at 37°C and 5% CO_2_. Images were acquired using Zen software. FRET imaging was performed as previously described (Kumar et al., 2016; Grashoff et al., 2010). Briefly, a 1-µm diameter ROI in the cellular midplane of the ER of fixed cells was imaged five to 10 times for eGFP fluorescence (ex. 488-nm laser, em. 527/55 filter) and TagRFP fluorescence (ex. 561-nm laser, em. 615/70-nm filter) prior to acceptor photobleaching using the 561-nm laser line. At least 10 sequential images taken 150–450-ms apart were acquired after bleaching for both donor and acceptor. The normalized FRET index was calculated as the ratio of the averaged donor intensity from the five images taken before bleaching and to the averaged donor intensity of the five images taken after bleaching: $1-I_{donor\;pre-bleach}/I_{donor\;post-bleach}$

### Recombinant protein expression and purification

For NOMO Ig expression, N-terminally His_6_-tagged Ig 1–2, Ig 1–2^3-Mut^, and Ig 10–11 proteins were each expressed in BL21(DE3) *Escherichia coli* strains. 4 liters cultures were grown in terrific broth at 37°C with shaking, and expression was induced at an OD_600_ of 0.7 with 1 mM IPTG for 4 h at 20°C. Cells were centrifuged at 8,000 × *g* for 30 min at 4°C frozen at −80°C until use. Pellets were resuspended in lysis buffer (50 mM Tris, pH 8, 50 mM NaCl, 2 mM 2-mercaptoethanol [βME], 10 mM imidazole, 1x Roche cOmplate EDTA-free Protease Inhibitor Cocktail, 30 µM PMSF, and 10% [vol/vol] glycerol), then subjected to three iterations of French pressure cell press. Following centrifugation at 20,000 × *g* for 30 min at 4°C, supernatants were incubated with Ni-NTA agarose (Qiagen) for 2 h with gentle shaking at 4°C. Samples were run through gravity flow columns and washed with 50 ml of wash buffer (50 mM Tris, pH 7.5, 300 mM NaCl, 5 mM MgCl_2_, 2 mM βME, 1x Roche cOmplate EDTA-free Protease Inhibitor Cocktail, and 25 mM imidazole). To elute, 5 ml of elution buffer (50 mM Tris, pH 7.5, 150 mM NaCl, 2 mM MgCl_2_, 1 mM βME, and 250 mM imidazole) was added to the column, and collected samples were assessed for purity by SDS-PAGE. Eluates were dialyzed overnight in 50 mM Tris, pH 7.5,150 mM NaCl, and 1 mM βME and then subjected to SEC with an S75 column. Samples were concentrated at 500 × *g* when needed using Amicon Ultra Centrifugal Filters.

For FL NOMO expression, Expi293F cells were transfected with either Flag-NOMO^WT^ or Flag-NOMO^4-Mut^ constructs using the ExpiFectamine 293 Transfection Kit (Gibco) following the manufacturer’s protocol for a 100 ml culture. Cells were harvested 72 h later and lysed in 60 ml of lysis buffer (50 mM MES, pH 6.0, 100 mM NaCl, 50 mM KCl, 5 mM CaCl_2_, 5% glycerol, and 1% n-dodecyl-β-D-maltoside [DDM]) for 1 h at 4°C. Lysates were spun for 30 min at 20,000 × *g* at 4°C, and the resulting supernatants were incubated with 0.5 ml anti-FLAG M2 beads (Sigma-Aldrich) overnight. Extracts were then loaded onto a gravity column and washed with 50 ml of buffer (50 mM MES, pH 6.0, 150 mM NaCl, 100 mM KCl, 5 mM CaCl_2_, 2% glycerol, and 0.05% DDM). To elute FLAG-tagged NOMO, beads were incubated for 30 min with 2 ml of elution buffer (50 mM MES, pH 6.0, 150 mM KCl, 5 mM CaCl_2_, 5 mM MgCl_2_, 2% glycerol, 0.05% DDM, and 15 μM FLAG peptide). The eluent was concentrated at 100 × *g* to 10 µM and subjected to SEC in an S200 or S75 column (GE Healthcare).

### SEC and SEC coupled with multi-angle light scattering

For in vitro–binding studies, His_6_-Ig 10–11 was incubated with either His_6_-Ig 1–2 or Ig 1–2^3-Mut^ in buffer containing 50 mM Tris-HCl, pH 7.5, 100 mM NaCl, 5 mM MgCl_2_, and 5 mM CaCl_2_. The mixture was incubated at room temperature for 1 h prior to running on an equilibriated Analytical Superdex-75 10/300 (Cytiva). His_6_-Ig 10–11 and His_6_-Ig 1–2^3-Mut^ binding were tested with an overnight incubation, as well as a 1:2 ratio of His_6_-Ig 10–11 and His_6_-1–2^3-Mut^. Apparent molecular masses were determined by calibrating the size-exclusion column with the following set of protein standards: thyroglobulin, ferritin, catalase, aldolase, albumin, ovalbumin, chymotrypsinogen, and ribonuclease A.

Multi-angle laser light-scattering experiments were performed at room temperature in 50 mM MES, pH 6.0, 150 mM KCl, 5 mM CaCl_2_, 5 mM MgCl_2_, 2% glycerol, and 0.05% DDM. Light-scattering data were measured using a Dawn Heleos-II spectrometer (Wyatt Technology) coupled to an Opti-lab T-rEX (Wyatt Technologies) interferometric refractometer. Prior to sample runs, the system was calibrated and normalized using the monomeric BSA as an isotropic protein standard. Samples at 1–1.5 mg/ml in 250 μl were injected into a Superdex 200 increase 10/300 Gl column (GE Healthcare) at a 0.5 ml/min flow rate. Data on light scattering (690-nm laser), UV absorbance (280 nm), and refractive index were measured simultaneously during the run. Data were processed in ASTRA software.

### Isothermal titration calorimetry assay

ITC measurements were executed on a VP-ITC Microcal calorimeter (Microcal) at 25°C. The buffer contained 50 mM Tris-HCl, pH 7.5, 100 mM NaCl, 5 mM MgCl_2_, and 5 mM CaCl_2_. All solutions were filtered and degassed. Starting with an upper cell analyte concentration of 300 µM His_6_-Ig 10–11—above which the fragment precipitated—we adjusted the titrant concentration to 20 µM for His_6_-Ig 1–2 and His_6_-Ig 1–2^3-Mut^. This minimum value ensured sufficient heat detection above the background, which was necessary due to the smaller enthalpic contribution compared with the entropic contribution of the interaction. Automated titrations injected 10–15 μl of His_6_-Ig 10–11 from a syringe into the sample cell containing either buffer, His_6_-Ig 1–2, or His_6_-Ig 1–2^3-Mut^ at a time interval of 120 s and repeated 22–33 times. The heat of interaction between both of the components was measured after subtracting the heat of dilution, and the baseline was subtracted. The titration data were analyzed by Origin7.0 (Microcal).

### Sequencing data

RNA sequencing data for control and DM1 tibialis anterior muscle biopsies is publicly available (GSE86356) and was published by Wang et al. (2019) (Transcriptome alterations in myotonic dystrophy skeletal muscle and heart). Raw reads were processed as previously described (Todorow et al., 2022). Normalized counts for NOMO1 were plotted against decreasing dorsiflexion strength as assessed by Wang et al. (2019) (Transcriptome alterations in myotonic dystrophy skeletal muscle and heart).

### Myoblast differentiation assays

C2C12 myoblast cell differentiation into myotubes was induced by shifting 100% confluent myoblasts from DMEM containing 10% FBS to media containing 2% (vol/vol) horse serum. Stable cell lines were generated in independent triplicates by lentiviral transduction and consisted of mixed populations. For differentiation experiments, cell lines were plated in 6-well tissue-cultured treated plates (Corning) or 10-cm^2^ plates at the same density and induced to differentiate upon confluency. Fixation and immunostaining were performed as described above. Fusion index was calculated by binning the number of nuclei per image according to whether they were absent or present within a Myhc-expressing myotube. Total nuclei and the number of nuclei that fused into myotubes were quantified per image (3× 20× and 5× 40× magnification). The fusion index was calculated as the ratio of fused nuclei to the total nuclei count, with a minimum criterion of three myonuclei per myotube, as previously described (D’Angelo et al., 2012; Gomez-Cavazos and Hetzer, 2015).

### *C. elegans* experiments

Nematodes expressing *myo-3p::myo-3::gfp* were maintained at 25°C. RNAi against *nra-4* was induced through feeding of *E. coli* HT115(DE3) expressing *nra-4* dsRNA obtained from the Ahringer RNAi library (Kamath and Ahringer, 2003). Nematodes were fed RNAi-containing bacteria and its respective control (HT115(DE3) expressing empty vector L4440) for two generations prior to the motility analysis. Nematodes were synchronized manually by egg laying. The motility of 4-day-old nematodes (young adults) was analyzed in M9 media. Nematodes were dispersed in fresh M9 media (750 μl in a 3.5-cm culture plate) and left to acclimatize for 30 s before video acquisition at 7.8-fold magnification and 25 frames per second. The videos were recorded in bright field using a Leica stereo microscope M205FA. 5–7 nematodes were recorded at once, and a total of at least 15–20 nematodes were recorded per replicate and per condition. Videos were analyzed using WormLab software (WormLab 2023.1.1 [MBF Bioscience LLC]). Body bends per second were calculated through the body angle-midpoint function of the software. Overlapping nematodes were censored from the analysis. Statistical analysis (one-way ANOVA) was performed using GraphPad Prism. P values were considered significant when: P ≤ 0.05 (*), P ≤ 0.01 (**), and P ≤ 0.001 (***).

### Statistics and reproducibility

Experiments were conducted at least three times, yielding consistent results, or three independent samples were analyzed to ensure data representativeness. The Shapiro–Wilk test was used to assess normality. Normally distributed datasets were analyzed using either a two-tailed unpaired *t* test or a one-way ANOVA. Non-normally distributed datasets were evaluated using a two-tailed unpaired Mann–Whitney test or a Kruskal–Wallis test. Statistical analyses were performed using GraphPad Prism 9.4.0, with significance set at P < 0.05. Data are presented as means with SD error bars. Sample sizes were not predetermined by statistical methods, and no data were excluded. Investigators were aware of group allocations during experiments and assessments, and randomization was not applied.

### Online supplemental material

Fig. S1 shows the AlphaFold prediction and conservation analysis of the NOMO Ig 1/10/11 interface. Fig. S2 shows that the NOMO depletion results in ER voids without perturbing global ER-resident protein diffusion. It also includes FRAP superplots. Fig. S3 shows that the replicate ITC results for Ig 1–2 and Ig 10–11 binding and co-immunoprecipitation of exogenous Ig 1–2 fragments with endogenous, WT, and 4-Mut NOMO. Fig. S4 shows the differential NOMO1 expression in myotonic dystrophy patients and correlation with muscle strength. It also shows that NOMO KO impairs myogenesis, and stable rescue of differentiation defects depends on the Ig 1/10/11 interface. Fig. S5 shows that the Nomo depletion mildly perturbs ER morphology and actin organization in myoblasts. Videos 1 and 2 show the compromised nematode motility upon nra-4 (NOMO homolog) RNAi treatment.

## Supplementary Material

SourceData F2 is the source file for Fig. 2.

SourceData F3 is the source file for Fig. 3.

SourceData F4 is the source file for Fig. 4.

SourceData F5 is the source file for Fig. 5.

SourceData FS3 is the source file for Fig. S3.

SourceData FS4 is the source file for Fig. S4.

## Data Availability

Source data for all graphical representations and unprocessed blot images are provided. All other data supporting the findings of this study are available from the corresponding author on reasonable request.
