# Supplementary figures and images for: Nodal modulator (NOMO) is a force-bearing transmembrane protein required for muscle differentiation
Source: J Cell Biol. 2025 Jul 15;224(9):e202505010. doi: 10.1083/jcb.202505010 (PMC12262048; doi:10.1083/jcb.202505010)

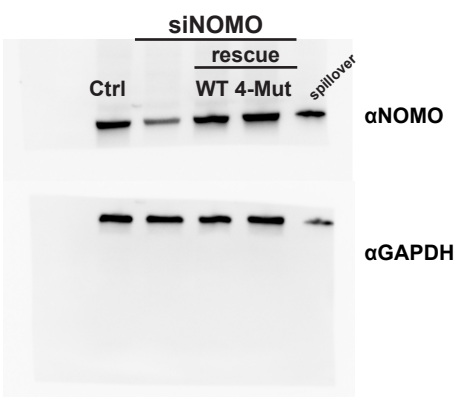

Supplement: SourceData F2 — is the source file for Fig. 2. [file jcb_202505010_sourcedataf2.pdf]

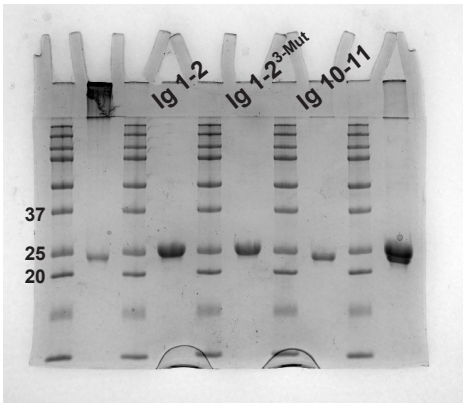

Supplement: SourceData F3 — is the source file for Fig. 3. [file jcb_202505010_sourcedataf3.pdf]

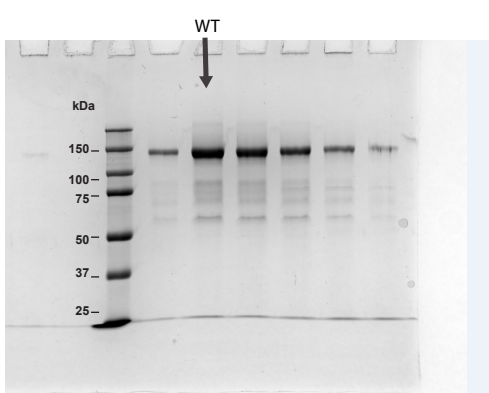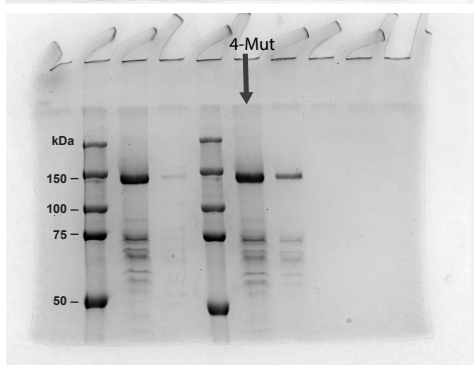

Supplement: SourceData F4 — is the source file for Fig. 4. [file jcb_202505010_sourcedataf4.pdf]

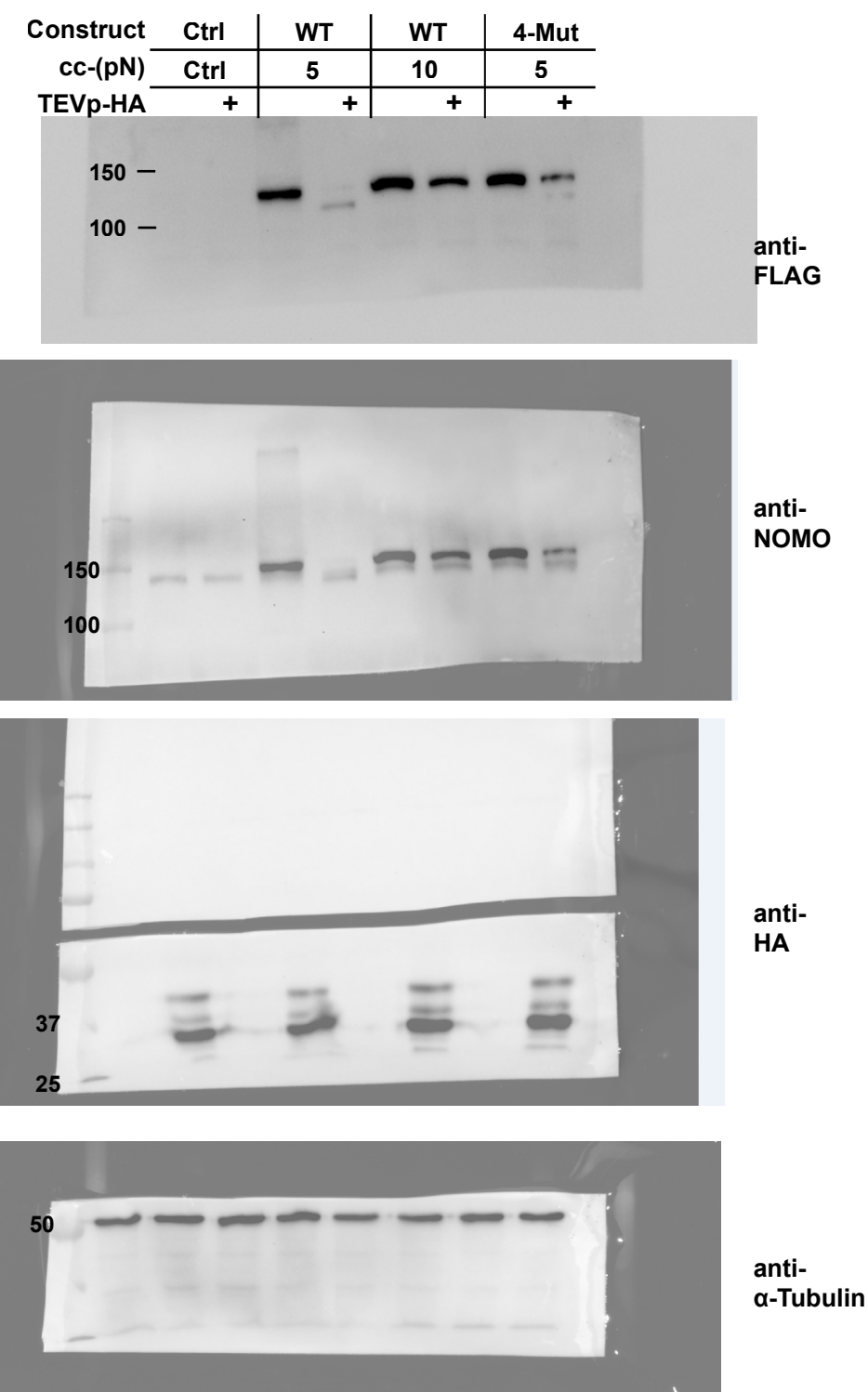

Supplement: SourceData F5 — is the source file for Fig. 5. [file jcb_202505010_sourcedataf5.pdf]

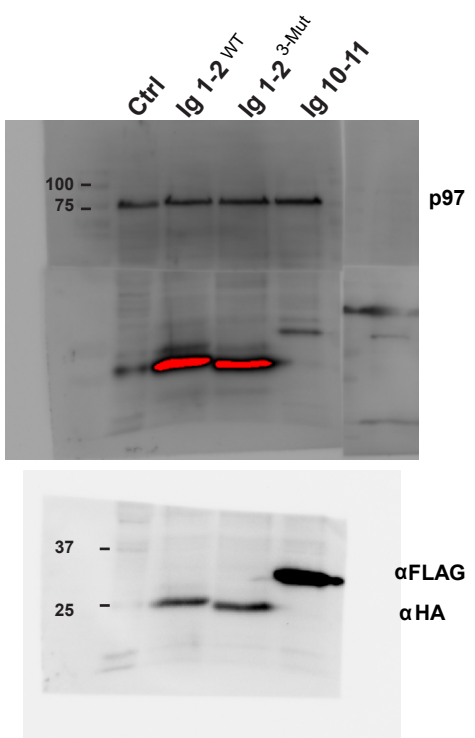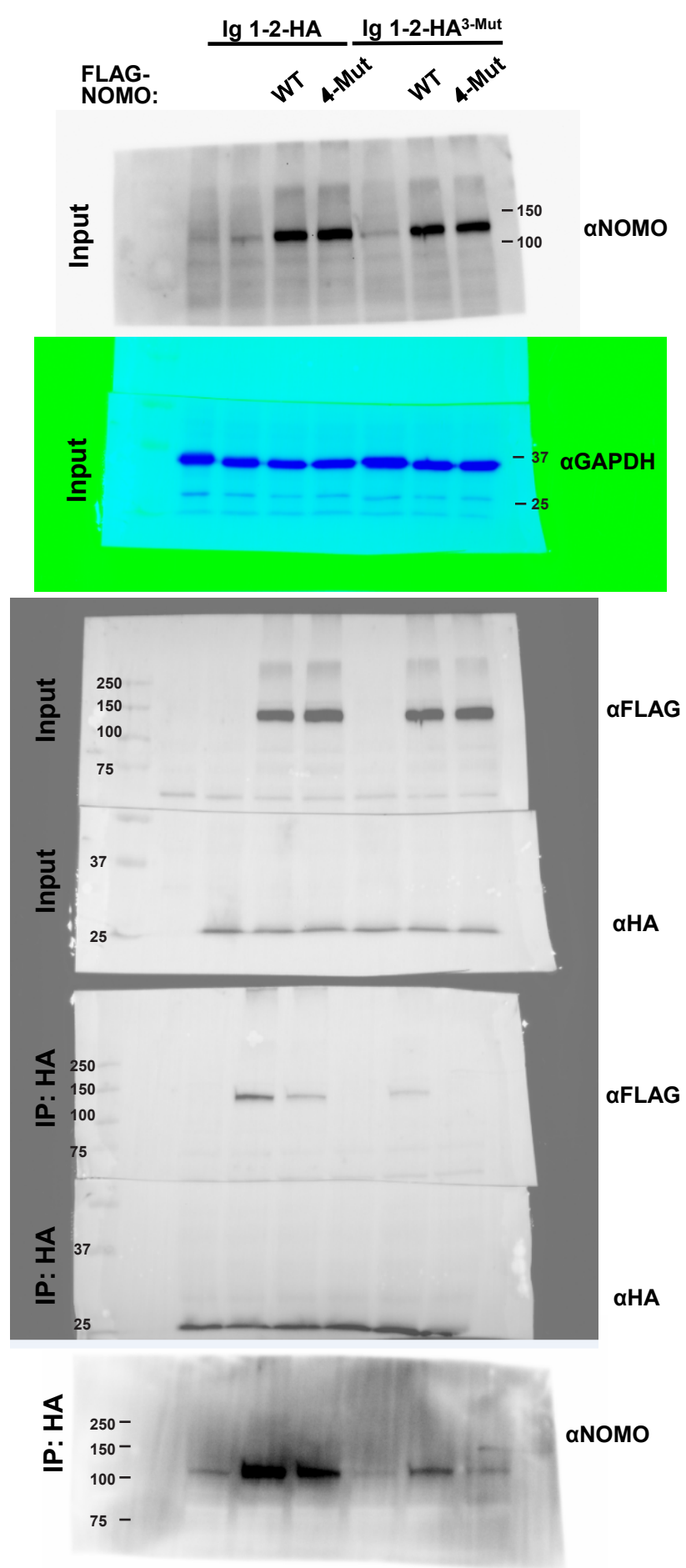

Supplement: SourceData FS3 — is the source file for Fig. S3. [file jcb_202505010_sourcedatafs3.pdf]

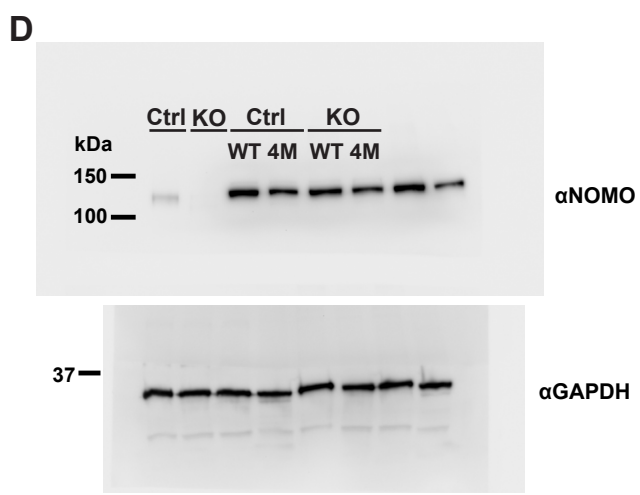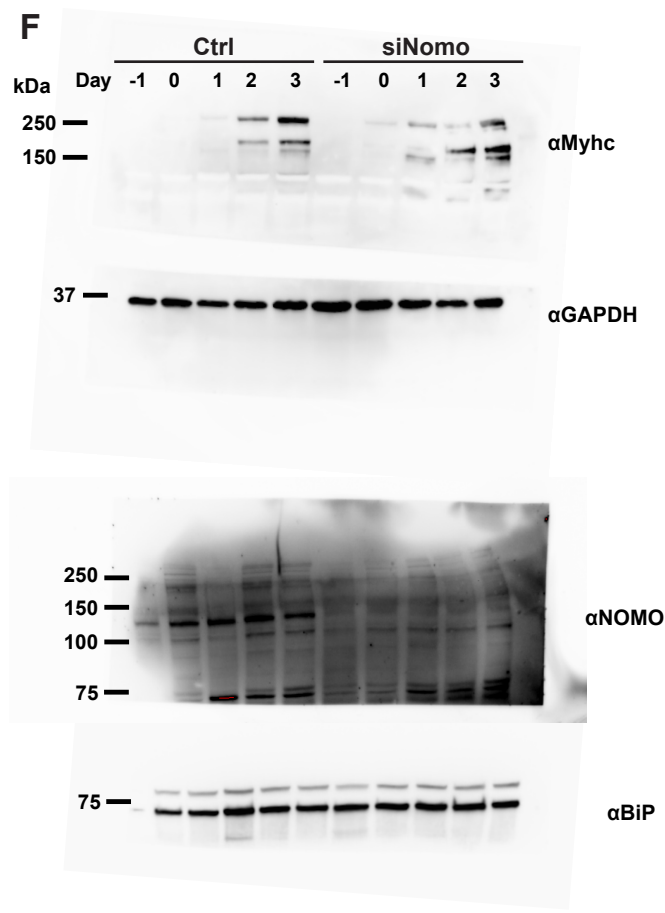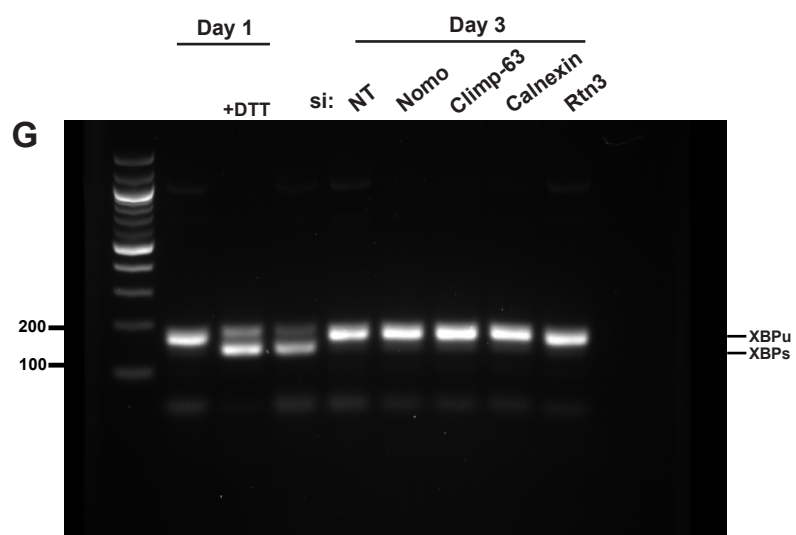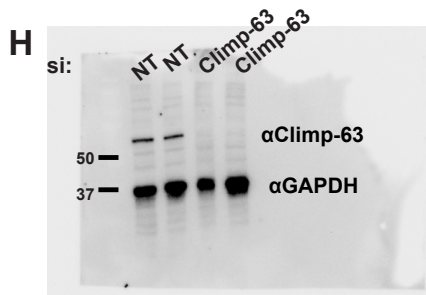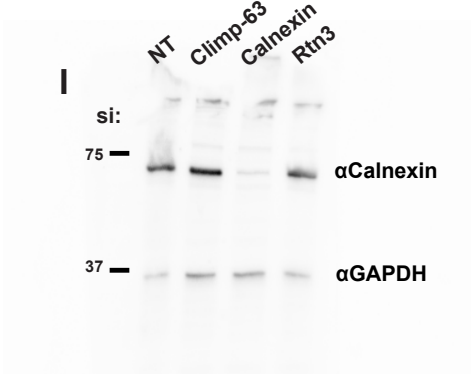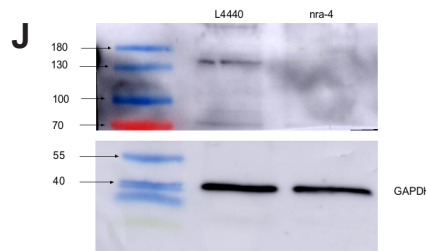

Supplement: SourceData FS4 — is the source file for Fig. S4. [file jcb_202505010_sourcedatafs4.pdf]
